# The glymphatic hypothesis: the theory and the evidence

**DOI:** 10.1186/s12987-021-00282-z

**Published:** 2022-02-03

**Authors:** Stephen B. Hladky, Margery A. Barrand

**Affiliations:** Department of Pharmacology, University of Cambridge, Cambridge, CB2 1PD UK

**Keywords:** Extravascular transport, Perivascular, Periarterial, Perivenous, Blood–brain barrier, Subependymal space, Glymphatic, Aquaporin 4, Bulk flow, Diffusion, Hydrophilic solute, Cerebrospinal, Interstitial, Fluid circulation

## Abstract

The glymphatic hypothesis proposes a mechanism for extravascular transport into and out of the brain of hydrophilic solutes unable to cross the blood–brain barrier. It suggests that there is a circulation of fluid carrying solutes inwards via periarterial routes, through the interstitium and outwards via perivenous routes. This review critically analyses the evidence surrounding the mechanisms involved in each of these stages. There is good evidence that both influx and efflux of solutes occur along periarterial routes but no evidence that the principal route of outflow is perivenous. Furthermore, periarterial inflow of fluid is unlikely to be adequate to provide the outflow that would be needed to account for solute efflux. A tenet of the hypothesis is that flow sweeps solutes through the parenchyma. However, the velocity of any possible circulatory flow within the interstitium is too small compared to diffusion to provide effective solute movement. By comparison the earlier classical hypothesis describing extravascular transport proposed fluid entry into the parenchyma across the blood–brain barrier, solute movements within the parenchyma by diffusion, and solute efflux partly by diffusion near brain surfaces and partly carried by flow along “preferred routes” including perivascular spaces, white matter tracts and subependymal spaces. It did not suggest fluid entry via periarterial routes. Evidence is still incomplete concerning the routes and fate of solutes leaving the brain. A large proportion of the solutes eliminated from the parenchyma go to lymph nodes before reaching blood but the proportions delivered directly to lymph or indirectly via CSF which then enters lymph are as yet unclear. In addition, still not understood is why and how the absence of AQP4 which is normally highly expressed on glial endfeet lining periarterial and perivenous routes reduces rates of solute elimination from the parenchyma and of solute delivery to it from remote sites of injection. Neither the glymphatic hypothesis nor the earlier classical hypothesis adequately explain how solutes and fluid move into, through and out of the brain parenchyma. Features of a more complete description are discussed. All aspects of extravascular transport require further study.

## Introduction

Compared to other organs in the body, the brain has much tighter control over what substances can enter and leave it. This control is made possible by the presence of a blood–brain barrier consisting of a tight endothelial layer lining the cerebral vasculature. The degree of access to the brain of solutes derived from the periphery will depend on their capability to cross this barrier and on the extent of blood flow. Distribution to within ca. 30 μm of brain cells by blood flow combined with transport across the blood–brain barrier and with diffusion the remaining short distance collectively comprise the intravascular route. Importantly for brain function and metabolism, O_2_, glucose, CO_2_, and H_2_O can be transferred rapidly in large amounts by this intravascular route as only it can supply and remove them in sufficient quantities (for references see [1, 2]). Many other solutes have specific transporters at the blood–brain barrier and although they do not cross in such quantities, they can nevertheless move by the intravascular route in sufficient amount to satisfy the requirements of the brain. But for a smaller group of endogenous solutes and many exogenous solutes including many putative drugs, transport across the barrier is inadequate to allow delivery or removal. It is now recognised that there has to be another, extravascular^1^ route for transport which avoids the blood–brain barrier. Transport of solutes by the extravascular route is important when neither metabolism within the brain nor transport across the blood–brain barrier will suffice for their supply or removal (for further discussion see Sect. 3.2 in [2], Sect. 4.3.4 in [3] and [4, 5]).

Solutes which depend upon the extravascular route for their transport are usually hydrophilic and thus cannot easily cross the membranes of microvascular endothelial cells. Spector et al. [6, 7] and Abbott et al. [5] discuss solutes, e.g. vitamin C and folate, that reach the parenchyma by extravascular entry from CSF and similarly Hladky and Barrand [2] compare and contrast efflux of many other solutes via the blood–brain barrier and via extravascular routes.

There is currently great interest in the possible involvement of extravascular elimination in the development of central nervous system disorders including: Alzheimer’s disease; idiopathic normal pressure hydrocephalus; Parkinson’s disease; Huntington’s disease; small vessel disease, traumatic brain injury and stroke [4, 8–34]. There can be little doubt that extravascular transport of solutes is an important process. This review considers the evidence for the idea that the mechanism for extravascular transport is a circulation of fluid as proposed in the glymphatic hypothesis.

**Fig. 1:**
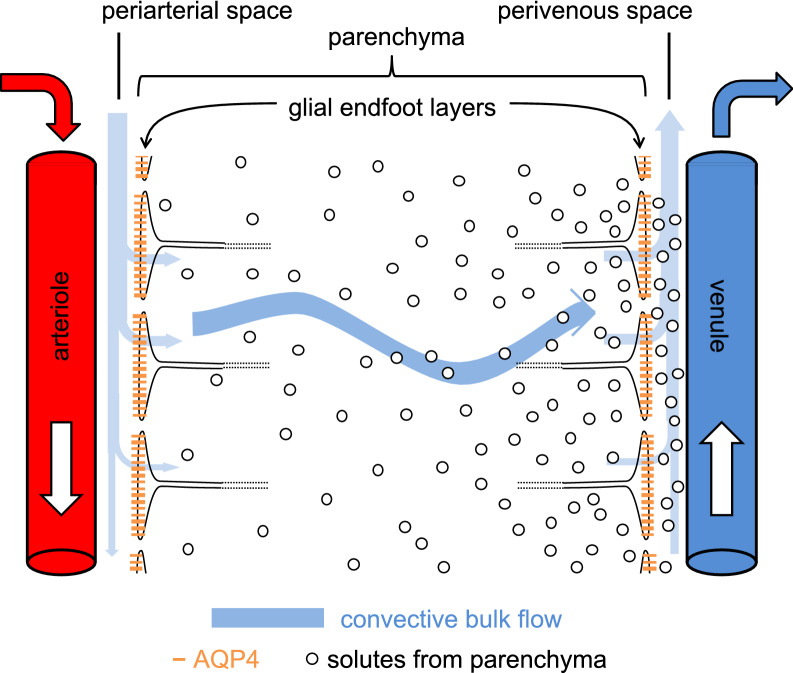
Principal features of the proposed glymphatic circulation. Periarterial inflow of cerebrospinal fluid, shown on the left, enters the interstitial fluid in the parenchyma by crossing a layer of glial endfeet assisted by the presence of aquaporin 4 (AQP4) water channels in the endfoot membrane facing the perivascular space. The fluid then flows through the interstitial spaces propelling solutes towards the perivenous conduits, shown on the right, leading to outflow from the brain. Flow in the perivascular space of a blood vessel is in the same direction as the blood flow, but the orientations of arterioles and venules vary and are not strictly antiparallel. Similar portrayals of the glymphatic hypothesis with varying artistic embellishments have been published repeatedly [11, 34, 36, 38, 42, 46, 238–241]. Above and in many other published figures flow is portrayed as sweeping solutes towards the venules where they become more concentrated. It is argued in Sect. 5.4 that such a sweeping effect is unlikely to occur

The word “glymphatic” as in “glymphatic hypothesis”, “glymphatic circulation”, “glymphatic system”, “glymphatic pathway” and “glymphatics” was introduced in 2012 [11]. The glymphatic hypothesis proposes that there is a circulation of fluid that is critical for the extravascular elimination of hydrophilic wastes from the brain (see Fig. 1). The circulation originates as CSF which flows into the parenchyma along periarterial spaces. The circulation continues through the parenchyma and then leaves via perivenous spaces. The proposed flow must cross the layer of glial endfeet that encase blood vessels within the brain firstly as it leaves the periarterial spaces into the parenchymal tissue and again as it enters the perivenous spaces. Both of these crossings are suggested to be facilitated by aquaporin 4 (AQP4) channels in the membranes of the glial endfeet. The assembly of components allowing the circulation is called the glymphatic system, the word glymphatics referring to the routes by which the flow occurs. The term “glymphatic” was coined because glial endfeet form the boundaries of parts of this circulatory system, its flow depending on the properties of these glial endfeet and its functions being related to that of lymphatics in peripheral tissues. The glymphatic hypothesis contains some attractive ideas such as there being:

- entry of a substantial portion of CSF into the interstitial spaces of the parenchyma, suggesting rapid delivery of nutrients and of drugs from CSF into the brain [11, 35];
- flow through the parenchyma providing an easily imagined mechanism for sweeping wastes into perivenous spaces [11, 36]; and
- rapid removal of metabolites and wastes from the parenchyma carried by flow along perivenous routes [11, 37].

The proposal of a glymphatic circulation for the delivery and removal of substances in the brain is a simple and seductive hypothesis. One testament to its popularity is that it has been discussed extensively in a number of reviews [2–5, 17, 20, 24, 30, 33, 34, 38–47] and a CrossTalk debate [48]. At the end of 2020 a search in Google Scholar on “glymphatic” produced 6860 results. Searching the more focused database of the Web of Science Core Collection produced 532 results with 147 in 2020. Searching the Web of Science for citations of the initial paper [11] showing evidence for the existence of a glymphatic circulation yielded 1513 results of which 268 were in 2020. Far from waning, interest in “things glymphatic” appears to be growing!

This review cannot cover all of the literature relating to glymphatics. However, it will provide an introduction to the concepts of the glymphatic hypothesis, will compare it with the earlier classical hypothesis for extravascular efflux of solutes and will consider the extent to which each of the hypotheses is supported by the available evidence.

## Background: Fluid and solute movements via extravascular routes

It had already been recognized by 1910 that spaces separated from the vascular lumen but associated with blood vessels coursing through the brain, so called perivascular spaces, were potential routes for fluid movements into and out of the brain parenchyma (for an extensive review of the history of the concept of “perivascular spaces” see [49]). In the early work it was shown that when pressure in the subarachnoid spaces was raised above normal, even carbon particles could be delivered along perivascular routes deep into the parenchyma [50, 51]. Inflow of fluid could also be seen without raised subarachnoid pressure. Thus, Weed [51] noted that after death when the vascular pressure drops to practically zero, the brain aspirates CSF. (This is explained using the principles underlying the Monro-Kellie doctrine [52].) Ma et al. [53] confirmed that there is a major influx of CSF markers into perivascular spaces immediately postmortem. Min Rivas et al. [54] noted the flow of CSF into the parenchyma that occurs immediately after cardiac arrest. Similarly Mestre et al. [26] found substantial influx of NaCl and other solutes from CSF in the early stages of severe ischaemic stroke. Furthermore, rapid inflow of CSF, which almost certainly has to be via perivascular spaces, was seen when fluid was withdrawn from the parenchyma into blood by making plasma hyperosmotic [55, 56]. However, while it is clear that such inflows can occur under unusual conditions, there is still controversy as to whether under normal in vivo conditions perivascular spaces within the parenchyma are inflated with free-flowing fluid.

###  Idea of perivascular spaces as preferred, normal exit routes. The classical hypothesis for extravascular efflux of solutes

For most of a century the consensus was that under normal conditions there was flow of fluid out of the parenchyma via “preferred routes” which came to include perivascular spaces, white matter tracts and subependymal spaces (see e.g. [5, 8, 10, 34, 49, 51, 57–64]. The driving force for this movement was thought to be a small hydrostatic pressure generated by fluid secretion across the blood–brain barrier (see. e.g. [57]) or more recently by some movements of the arterial walls [65]. The outward flow was thought to serve as a route for the elimination of wastes that cannot cross the blood–brain barrier and evidence was presented suggesting efflux of substances like serum albumin, polyethylene glycols and dextrans by periarterial routes. Exit of these hydrophilic substances from the parenchyma was too fast to be accounted for by diffusion alone, thus some form of flow (advection) had to be involved. The most common idea, which can be called the classical hypothesis, was that solutes moved through interstitial spaces by diffusion, but along the “preferred routes” by flow plus diffusion, i.e. convection. Some workers even considered an onward connection via arterial walls to true lymphatics [63, 66, 67]). As described in Sects. 5.2.2 and 5.5, the evidence on which each part of the classical view of extravascular efflux of solutes was based still stands (for reviews and extensive referencing see [3, 5, 59, 62, 63, 68, 69]).

### Idea of perivascular spaces as normal entry routes

However, repeatedly there were publications suggesting that the prevailing view needed modification and that perivascular spaces provided routes of entry for solutes and markers, for example: carbon black particles [70]; serum albumin [71]; sucrose, inulin and serum albumin [72]; and horseradish peroxidase [73–78].

The results reported for penetration of horseradish peroxidase by Rennels et al. [75, 79] following infusion into a lateral ventricle of a cat were striking. Using a particularly sensitive assay they observed that uptake of the peroxidase occurred into the cortex within 6 min which is approximately the time taken for the infused fluid to reach the adjacent subarachnoid spaces. This meant that penetration from the subarachnoid space into the parenchyma was effectively instantaneous! One suspects there was disbelief by other investigators that penetration could be so fast and that this was a major factor in promoting continued scepticism. There was also the elegant demonstration that penetration of solutes from the ventricles into the parenchyma was slow and diffusive [80], a result that has subsequently been confirmed by others [11, 81]. Scepticism about rapid penetration was seemingly justified by a report observing only slow back-and-forth movements of carbon black particles along exposed cortical surfaces, which did not support the idea of rapid influx [82].

An explanation for the apparently instantaneous initial uptake seen by Rennels et al. may be provided by the post-mortem uptake of CSF into the perivascular spaces [51, 53]. The uptake into the parenchyma from the adjacent subarachnoid space may have occurred in the time between killing the animal and obtaining specimens for examination. It would then follow as Rennels et al. noted [75] “The regional variability in tracer distribution, both along the pial surface and outlining the intraparenchymal microvasculature … was thus consistently related to probable local differences in [horseradish peroxidase] concentration in the subarachnoid space.“ This leaves open the question of the rate of penetration of horseradish peroxidase during life.

With some exceptions, notably the review by Abbott in 2004 [69], acceptance of the idea that there is normally influx of strongly hydrophilic solutes via perivascular spaces had to await evidence of solute movements measured in real-time whilst the animals were still alive.

## The original evidence of periarterial solute movements into the parenchyma in real-time in vivo

Publication of papers in 2012 and 2013 utilizing two-photon and conventional fluorescence microscopy [11, 37, 83] and magnetic resonance imaging (MRI) [84] established two points beyond reasonable doubt:

- In vivo there can be movements of a range of sizes of hydrophilic solutes from CSF into perivascular spaces surrounding arteries that penetrate the parenchyma.
- For the smaller solutes there is entry into the surrounding parenchymal interstitium.

These and other results from the seminal papers [11, 37, 83, 84] are summarized as follows:

- When added to artificial CSF perfusing the ventricles, FITC-dextrans were seen to enter only minimally into the parenchyma within 30 min in agreement with the earlier study of Rall [85] which found that solute entry into grey matter from the ventricles was slow and by diffusion.
- By contrast when added to CSF in the cisterna magna by 5-min infusion, FITC-dextrans spread within a few minutes along arteries in the subarachnoid space over the dorsal cortical surface and over 10’s of minutes down into the cortex via periarterial spaces. The smaller 3 kDa and 70 kDa dextrans spread from the surface arteries into the immediately adjacent cortex and from the perivascular regions along the penetrating arteries into the neighboring parenchymal tissue^2^. 2000 kDa dextran spread into the perivascular regions around penetrating arteries but did not enter the interstitial fluid of the parenchyma. These in vivo results were consistent with the distribution of the dextrans determined at a number of time points using standard histological methods. The histological results also indicated substantial entry of the smaller dextrans from ventral surfaces of the forebrain.
- Looking at smaller dextrans that entered from the dorsal surface, their fluorescence could be detected first in periarterial spaces, then in the parenchyma and finally in perivenous spaces of larger veins. These observations are consistent with the idea of a circulation of fluid reaching these locations in sequence. A similar time sequence was seen earlier with horseradish peroxidase by Rennels et al. [76]. Furthermore, Pizzo et al. [86] have since reported that antibodies infused into CSF appear along periarterial spaces well before being seen along perivenous spaces. The time taken for the progression of the markers from periarterial to perivenous spaces is broadly consistent with the time course of removal from the parenchyma of hydrophilic substances that cannot cross the blood–brain barrier^3^ (reviewed in [2]).
- The MRI study of Iliff et al. [84] showed that 938 Da and 200 kDa paramagnetic contrast agents (Gd-DTPA and gadospin respectively) spread from the cisterna magna along the course of arteries running within subarachnoid spaces. There was penetration of the lower MW agent into the parenchyma. However, as MRI has lower spatial resolution and less sensitivity for detection of the paramagnetic agents compared to the two-photon detection of the fluorescent probes, the volume of tissue showing detectable amounts of Gd-DTPA was substantially less than that in the studies using low molecular weight fluorescent dextrans discussed in the previous bullet point. As noted by the authors, this implies that the concentrations of both the dextrans and Gd-DTPA in the parenchyma were substantially less than along the surface arteries.
- Absence of AQP4 had effects on the movements of markers. The rates of both influx into the parenchyma of the smaller fluorescent dextrans and an albumin and the efflux of [^3^H]-inulin and 10 kDa [^3^H]-dextran injected directly into the parenchyma were substantially reduced in AQP4 (−/−) knockout mice compared to wild type. However, entry of all of the dextrans into the perivascular spaces of the penetrating arteries was maintained.

The results of the studies by Iliff et al. described above established that influx of markers from CSF to the parenchyma does occur though substantially more slowly than that reported earlier for horseradish peroxidase by Rennels et al. [75]. To provide further interpretation of their results Iliff et al. [11] introduced the glymphatic hypothesis.

## The development of the glymphatic hypothesis

**Fig. 2:**
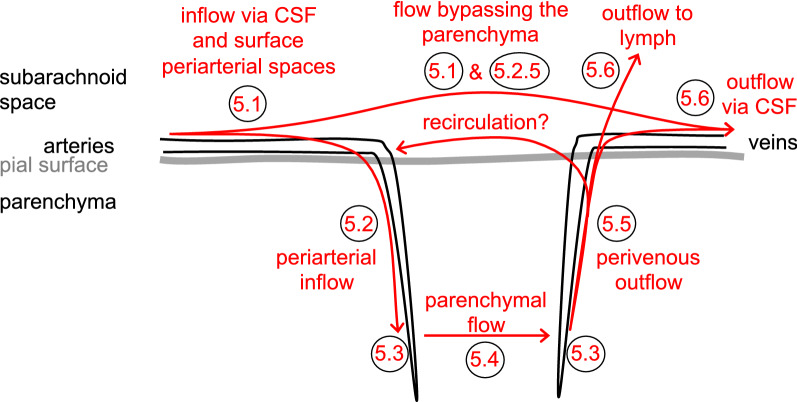
Diagram illustrating stages of fluid circulation considered in the discussion of the glymphatic hypothesis. Circled numbers refer to the sections in this review where the stages are considered in detail

The glymphatic hypothesis, introduced to explain the results found by Iliff et al. [11], stated that there is a circulation of fluid (see Figs. 1 and 2) which can be considered to occur in five stages, these being:

- fluid flow inwards from brain surfaces via periarterial spaces (in Fig. 2 marked as Sect. 5.2);
- transfer of the fluid from the periarterial space into the parenchymal interstitium with some of the water passing through AQP4 in the endfoot membrane facing the endothelial cells (marked as Sect. 5.3);
- flow through the interstitium described as a flow sweeping wastes towards perivenous spaces (marked as Sect. 5.4);
- transfer of fluid out of the parenchymal interstitium into the perivenous spaces with some of the water passing through AQP4 (marked as Sect. 5.3);
- flow outwards via perivenous spaces to subarachnoid spaces and/or to lymph (marked as Sects. 5.5 and 5.6).

In addition, when interpreting experimental results it is important to consider the extra initial stage (in Fig. 2 marked as Sect. 5.1) in which CSF must flow from the sites of administration of markers or tracers to the entry sites into the parenchymal periarterial spaces. Furthermore it is important to consider that a substantial proportion of the CSF does not enter the parenchyma (marked as Sects. 5.1 and 5.2.5).

Note that in the steady-state, the glymphatic hypothesis requires that periarterial inflow, net flow through the parenchyma and perivenous outflow must be the same -- there can be no accumulation or depletion of fluid in the perivascular spaces or interstitium, otherwise the volumes would be changing.^4^ This hypothesis takes no account of fluid derived from secretion across the blood–brain barrier.

Periarterial inflow was proposed to account for observations of the inward movement of fluorescent solutes occurring adjacent to arteries and at a rate faster than could possibly be achieved by diffusion alone. Perivenous outflow, which had already been suggested by others [75, 87, 88]), was proposed to provide a route for removal of solutes from the parenchyma and to explain how certain fluorescent solutes subsequently reached the walls of large veins. Flow through the parenchyma was postulated both to connect inward and outward flows and to sweep wastes generated in the parenchyma to the perivenous spaces and thence out of the brain (see Fig. 1).

The hypothesis as originally stated proposed that fluid is pumped along the periarterial spaces by cyclic changes in diameter of the arterioles which compress and enlarge the periarterial spaces propelling fluid forward by a sort of peristalsis [11, 36, 83, 89]. This periarterial inflow would increase pressure in the parenchyma thus providing a driving force for flow through the interstitial spaces and for outflow by the perivenous spaces. The perivenous outflow would also be aided by cyclic changes in diameter of the veins.

In some versions of the glymphatic hypothesis the perivenous outflow is thought to enter the subarachnoid spaces where it mixes with CSF (e.g. [36, 76, 90, 91]. These versions can easily be modified and extended to include outflow via white matter tracts leading to the ventricles [60, 64, 92].

In alternative versions, routes are proposed for at least some of the outflow leading to cervical lymphatics or to lymphatics in the dura [11, 93, 94, 95]. These versions echo previous proposals that the brain contains pre-lymphatics that serve to direct wastes into the lymphatic drainage from the head [66, 67, 96].

The following sections will consider the evidence for and against the glymphatic hypothesis.

## Detailed consideration of each of the stages described in the glymphatic hypothesis

The glymphatic hypothesis proposes a circulatory fluid flow. It is important to note that the flows within parenchymal perivascular spaces and interstitium invoked in the hypothesis are very difficult to measure by any direct means. It is thus not surprising that with the sole exception of the CSF inflow which contributes to volume changes in the parenchyma at the onset of ischaemic oedema [26] this has not been done. Tracers for water cannot be used as flow indicators because they escape from the flowing fluid (see e.g. [3]). Instead flows have been estimated from fluxes of markers assuming that these are carried with the flow and that any perceptible movements for any other reason are explicitly taken into account. When applied to CSF moving over large distances and with carefully chosen markers, these assumptions appear to be valid. However, it must be borne in mind that within the perivascular and interstitial spaces of the parenchyma, these assumptions may not be valid.

### Evidence of hydrophilic solute fluxes and flow along periarterial spaces within the subarachnoid space

Evidence for the glymphatic hypothesis has come from studies in which appropriate markers have been administered into CSF either in the cisterna magna, a lateral ventricle, or the intrathecal space of the spinal cord and their emergence onto the surface of the cortex followed. Solutes infused into the cisterna magna must travel through the cisterns and subarachnoid spaces to reach the observation sites, often the points of entry and exit of blood vessels supplying the cortical parenchyma on the dorsal surface. It is thought that these vessels are somehow involved in the movement of these solutes.

To reach the dorsal surface of the cortex the arteries traverse the ventral cisterns and then run through the subarachnoid spaces along the pial surface (see e.g. Fig. 2 in [97]). In their studies with mice Iliff et al. [11] observed that the fluorescent dextrans administered into the cisterna magna travelled rapidly along the subarachnoid arteries and then, less rapidly, spread out into the surrounding CSF in the subarachnoid space as well as following the branches of the arteries penetrating the cortex. In another study using MRI Iliff et al. [84] demonstrated that there was also rapid transport in CSF along ventral surfaces of the forebrain.

**Fig. 3:**
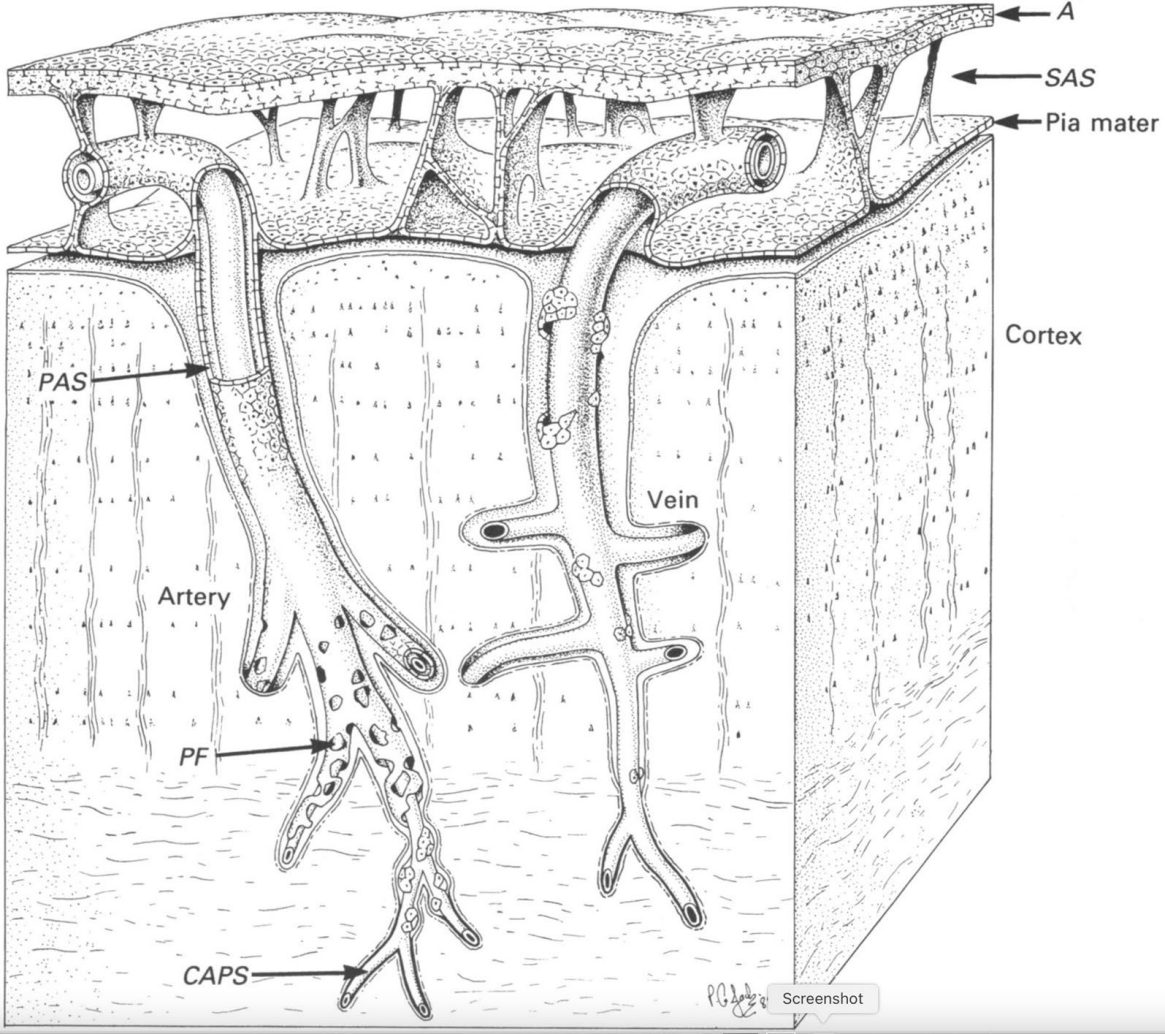
Meningeal layers associated with a cortical penetrating artery and an emerging vein. This is based on evidence obtained using electron microscopy. Note that the artery has a pial sheath as it courses along the surface of the cortex and this sheath follows the artery without break as it penetrates the cortex. By contrast the sheath around the emergent vein is not present along the course within the cortex. A, arachnoid membrane; SAS subarachnoid space (which on the dorsal surfaces of the cortex may be collapsed other than where it covers a blood-vessel); PF pial perforations. For more recent discussion of the presence (arterial) or absence (venous) of a pial sheath within the parenchyma see [127]. Reproduced with permission from Zhang et al., J. Anat. 1990 [100]

The exact way the markers travel along the vessels at the dorsal surface has been a matter of dispute. Iliff et al. [11] proposed that the fluid moved in a periarterial space contained within a pial sheath such as that shown in Fig. 3. However, they did not explain how the markers might enter this space (see [38]) or how the rapid flow through the space was reconciled with the much slower entry into the parenchyma along the penetrating arteries. Presumably entry into the periarterial spaces of markers added to CSF occurs in the basal cisterns where CSF from the cisterna magna first encounters the arteries. This entry may occur via stomata found in the pial sheath of all arteries inspected in the subarachnoid space [86]. These stomata could also allow markers to exit the sheath to spread out into the surrounding CSF. Bedussi et al. [98, 99] put forward a counter-view that no sheath is needed to explain the observations because the subarachnoid space on the dorsal surface of the brain is collapsed everywhere except in the periarterial regions. Their data and the firm evidence from electron microscopy for the presence of a sheath (see [100], though possibly not in the spinal cord [101]) can be reconciled if the sheath is somewhat permeable to both solutes and water as proposed by Pizzo et al. [86].

**Fig. 4:**
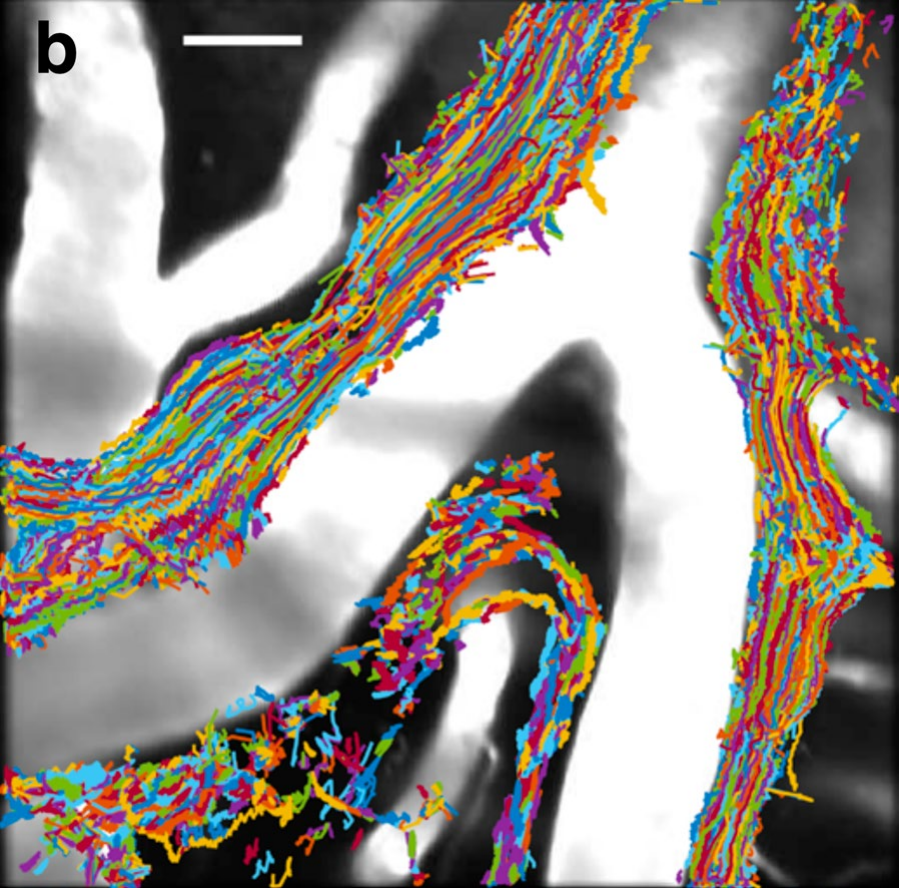
Trajectories of microspheres overlaid onto an image of surface blood vessels in the subarachnoid space. The trajectories are clustered along and parallel to the surfaces of arteries as if they are restricted to a periarterial space. Scale bar: 40 μm. (Reproduced from Mestre et al., Nature Comm. 2018 [89] Creative Commons Attribution 4.) From the relative straightness of the trajectories and the profile of velocities, maximum half-way across the width of the periarterial space, it has been concluded that periarterial spaces within the subarachnoid space are occupied by free fluid rather than by a porous matrix or gel [54]

Evidence that periarterial spaces surrounding subarachnoid arteries are used as conduits for fluid flow and solute movements also comes from experiments tracking the course of microspheres added to CSF in the cisterna magna. Pulsatile back and forth movements of the microspheres in time with the cardiac pulse were seen. Furthermore when the trajectories of these were plotted on top of an image of the blood vessels, they were alongside the arteries (see Fig. 4) [89, 99, 102]. The average displacement of the microspheres was in the same direction as the blood flow implying that there is a net flow of fluid along these periarterial spaces. Similar evidence has been obtained in studies using other markers including India ink [70, 97], gadolinium contrast agents [103, 104] and fluorescent molecules [11, 53] strengthening the belief that there is periarterial flow in the same direction as blood flow along subarachnoid arteries.^5^

There are two alternative interpretations of the observations in the microsphere experiments described above concerning destinations of the fluid and microsphere movements within the sub-arachnoid space. Bedussi et al. [99] were of the opinion that most of the net flow of CSF along the subarachnoid perivascular space proceeded to sites of outflow from the brain, in particular the cribriform plate, without entering the parenchyma. By contrast Mestre et al. [89] described their results as if most of the flow in the subarachnoid perivascular spaces continues within a sheath into the periarterial spaces surrounding penetrating arteries. (It will be argued later in Sect. 5.2.2 that most of the flow must be directed into the parenchyma if a glymphatic circulation is to account for the elimination of wastes.) On present evidence, with a leaky sheath there is no requirement for flow in the subarachnoid periarterial spaces to be equal to that in the parenchymal periarterial spaces (compare [44] and the commentaries by Bakker and van Bavel and by Kurtcuoglu et al. in [48]). It must always be borne in mind that careful argument is required before results obtained for movements in either parenchymal or subarachnoid periarterial spaces can be extrapolated to the other.

The exact mechanism by which CSF movements are driven at cardiac frequency within the subarachnoid periarterial spaces is still uncertain. The possibilities are local changes in the diameter of the arteries as proposed by Mestre et al. [89] or the much more general changes in brain vasculature that drive pulsatile CSF flows through the cerebral aqueduct and foramen magnum ([105–107] and for discussion [3, 38]). Kedarasetti et al. [108] modelled the possibility of peristaltic flow in subarachnoid periarterial spaces and concluded that any realistic changes in arterial diameter would be too small to drive any observable net flow. They found that effective peristaltic pumping would require changes in the dimensions of the perivascular spaces to be near 50 %. The calculations by Kedarasetti et al. thus favour the more general mechanism. (For further discussion of flows in periarterial spaces see [109–111].) Regardless of what drives the movements, from the patterns of microsphere trajectories (see Fig. 4), the spread of fluorescence within the subarachnoid spaces seen with dextrans, and the water movements seen with the long echo time diffusion weighted MRI [112], the pulsations and net movements in subarachnoid periarterial spaces must be considered to be flow processes.

While at least some of the larger veins are in contact with CSF, whether there is generally delivery of hydrophilic solutes to the mouths of parenchymal perivenous spaces is unclear. Reports vary. Zhang et al. observed delivery of carbon black particles into perivascular spaces of both arteries and veins when India ink was injected into the subarachnoid space over the vertex of the left frontal lobe. Iliff et al. [11] and Mestre et al. [89], who observed events at the dorsal surface of the brain, found delivery limited to periarterial spaces where the arteries penetrate into the parenchyma. By contrast Bedussi et al. [98] viewing events at the ventral surface saw delivery of large fluorescent dextrans close to sites of penetration of arteries and emergence of veins. They saw no access of the dextrans to the spaces around penetrating vessels, arteries or veins, on the dorsal surface. Ma et al. [53] found delivery along dorsal surface arteries and veins in anaesthetized but not in awake mice. Thus the available data are inconsistent.

There is substantial evidence for large variations in the delivery of solutes by CSF fluid movements. When radio-iodinated serum albumin was administered to awake patients intrathecally or intracisternally in sufficient dose and followed using cisternography, the tracer was seen to reach the basal cisterns and then spread over the cortex. Subsequently, when most of the tracer had left the brain, the amount of tracer remaining near the top of the brain was greater than that seen towards the bottom [113–115]. The same behaviour was seen but at higher resolution using MRI with cisternal administration of gadolinium contrast agents in humans [116, 117] and rats. In rats the agents travelled from the cisterna magna along the ventral surface of the brain to the olfactory bulb [118, 119] with prominent spread along the divide between the cerebellum and cerebrum, the middle cerebral artery and the rhinal fissure. Large areas of the cortical surface saw very little of the contrast agent. In all of these studies concentrations initially were much higher on the ventral surfaces than on the dorsal surfaces, while later the relative concentrations were reversed.^6^

Delivery of markers to dorsal cortical surfaces was also affected markedly by the rate and detailed method of their infusion into the cisterna magna. Smith et al. [120] suggest that this explains much of the variation found by various groups in entry rates into the parenchyma when markers were added cisternally.

The observed movements of markers carried by CSF are also affected by anaesthetics. Ma et al. [53] compared delivery of extracellular fluid markers from the ventricles to the dorsal subarachnoid spaces in mice that were awake or anaesthetized with either isoflurane or ketamine/ medetomidine. In awake mice there was rapid elimination of most of the dose, primarily via perineural routes including the olfactory nerve crossing the cribriform plate, with very little reaching the dorsal subarachnoid spaces. By contrast with the anaesthetized mice the marker was removed from the brain much less rapidly and a significant amount was delivered to the dorsal subarachnoid perivascular spaces.

Anything which changes the distribution of CSF flow can have large effects on entry of markers into the parenchyma in a specific region, in particular the cortex accessible for two-photon microscopy just below the dorsal surface (see e.g. [121]). A redistribution of CSF flow (or subarachnoid periarterial flow) away from the dorsal surfaces of the cortex (as in [53]) would provide a plausible explanation (as is needed, see [18]) for much of the 20-fold smaller delivery of fluorescent markers to the parenchyma when mice were awake rather than asleep [37]. More generally such differences should be taken into account whenever penetration into the brain of markers added to the cisterna magna, ventricles or intrathecal spaces of the spinal cord is being interpreted.^7^

### Hydrophilic solute fluxes and fluid flow along periarterial spaces in the parenchyma

The glymphatic hypothesis proposes that solutes in the subarachnoid periarterial space continue into the parenchyma along these spaces, but do not flow out by the same route. However, there is good evidence that both inward and outward movements of solutes can in fact occur along periarterial pathways. Indeed as Bakker et al. [17] put it “in most studies in which parenchymal injections of tracers were used, it was concluded that [periarterial] flow is outward … while in the studies in which tracers were injected into the cisterna magna, inward flow was observed …” (see also Sect. 4.3.4 in [3]).^8^ Of course, in both types of study the actual measurements were of solute movements from which fluid movements were inferred. In all cases the solute fluxes observed were in the direction favoured by a large concentration gradient of the solute. This casts doubt on the idea that the movements were carried by a net flow of fluid.

#### Evidence of periarterial influx of hydrophilic solutes

Many studies in addition to those cited in Sect. 2.2 (see e.g. [122]) now support the idea that periarterial influx of solutes occurs in vivo. However, the impressions given by different authors concerning the rate and extent of this influx vary widely. Contrast the accounts in [76] for very fast influx, [11, 84] for fast, extensive influx, [103, 117, 118, 123, 124] for more modest influx, [125] and [53]^9^ for slow or non-existent influx, and [44, 126, 127]^10^ for possibly artefactual influx depending on pressure produced by infusion of the markers. Solutes in addition to those mentioned in Sect. 2.2 reported to be able to use extramural perivascular pathways to gain entry to the parenchyma include: fluorescent dextrans [11], MRI contrast agents, e.g. Gd-DPTA and gadobutrol [84, 117, 118, 128], amyloid-β [129], 15 nm nanoparticles [130] and NaCl [26, 45, 93]. It has yet to be shown that periarterial influx can lead to clinically useful delivery of therapeutic agents ([131–133] but see [86, 134–137]).

#### Evidence of periarterial efflux of solutes

There is strong evidence to support the possibility of periarterial efflux of solutes occurring from the parenchyma.

- The “classical” evidence for periarterial efflux was presented in research from Cserr, Bradbury, and colleagues initially using serum albumin but then also horseradish peroxidase. Bradbury, Cserr and Westrop [138] found that when ^125^I-albumin was injected via an indwelling guide cannula (inserted a week before) into the caudate nucleus on one side of the brain it was subsequently seen at high concentrations in the walls of the ipsilateral arteries connecting the circle of Willis to that region. These concentrations were much higher than those in either CSF outside the arteries or in the walls of the contralateral arteries, which excludes the idea that the albumin arrived in the arteries via the CSF. Szentistvanyi et al. [62] extended these results by studying the distribution of injected Evan’s blue labelled albumin and horseradish peroxidase, finding these markers located prominently along the outside of major arteries supplying the regions of injection. The only plausible explanation is that under the conditions of their experiments the albumin reached the arteries by a periarterial pathway. Studies by Yamada et al. [96] provided similar results further confirming the plausibility of efflux via periarterial routes.
- More recently using fixed sections and high resolution microscopy, Weller, Carare and colleagues [8–10, 129, 130, 139] found that after a short delay dextrans, ovalbumin and amyloid-β injected into the parenchyma were found primarily within the smooth muscle coat along the walls of arteries but were not found along veins. When added to CSF, amyloid-β initially was found along the outer surface of arteries, but subsequently within the smooth muscle as if it first had to enter via an extramural route before it could exit via an intramural route (see next section).^11^ The distribution of labelled amyloid-β injected into the parenchyma was similar to that of the amyloid deposits seen in cerebral amyloid angiopathy suggesting that the extravascular efflux route for amyloid-β was in fact along arteries [4, 8, 129, 140].
- During infusions of Evan’s blue labelled albumin into the inferior colliculus Ball et al. observed the albumin along the middle cerebral artery [141] and in the walls of ipsilateral arteries as far as the circle of Willis [142].
- Arbel-Ornath et al. [12] using in vivo two-photon imaging of 3 kDa cascade blue dextran injected into the cortex found the dye rapidly accumulated along arterial walls but not veins.
- Liu et al. [143] found that fluorescently-labelled ovalbumin injected in the parenchyma of grey matter in the spinal cord moved radially outwards in perivascular spaces along both arterioles and venules.

These results may be challenged to varying extents on the basis that they are artifacts of the altered local hydrostatic pressure produced by the infusions. Thus elevated pressures might selectively collapse some efflux pathways and would inevitably drive efflux by the pathways of least resistance. It is certainly true that even the smallest volumes and lowest rates of infusion that have been used represent large but local perturbations of the tissue (even in rats and much more so in mice).^12^ That infusions can produce outflow from a region has been exploited in studies on convection-enhanced drug delivery [144]. However, the duration of infusion and the infused volumes in the drug delivery studies were much larger than those in the studies on marker efflux listed above. Furthermore the infusion rates used for drug delivery were more than 8 fold larger than those employed by Bradbury, Cserr and coworkers. In addition, it is likely that the pressure disturbances caused by an initial infusion are short-lived compared to the hours over which efflux was determined in their studies on efflux from the parenchyma (see footnote 12). (That efflux was described by a single rate constant [62] i.e. it apparently occurred with an unchanging mechanism.) All the data obtained in the studies in the list above provide evidence that there can be efflux of solutes by periarterial routes while those from the classical studies from Bradbury, Cserr and coworkers provide strong evidence that periarterial efflux occurs normally. The data from Weller, Carare and coworkers suggest that a periarterial pathway is important for efflux of amyloid-β, which is, of course, a waste product of great clinical interest.

#### Proposed routes for periarterial influx and efflux of solutes

**Fig. 5:**
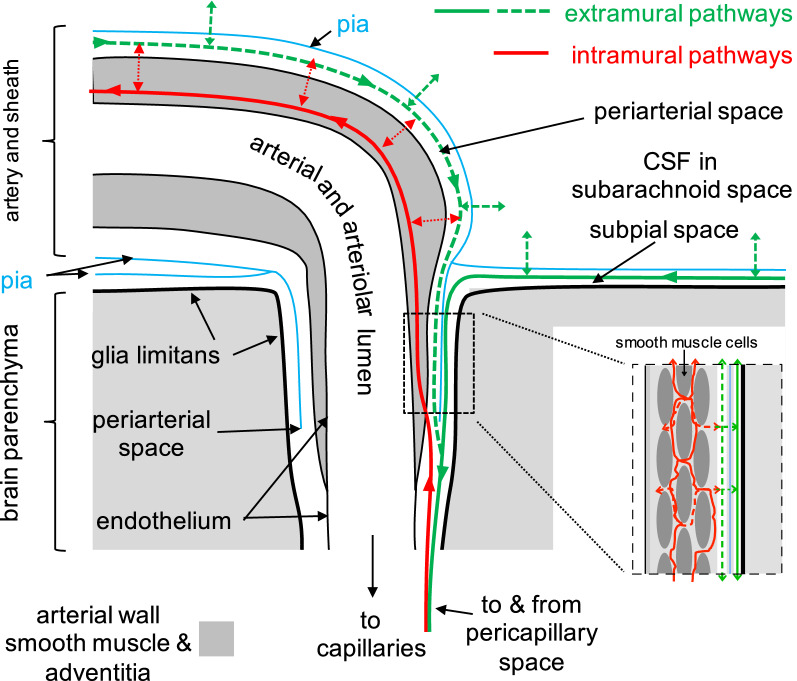
Possible routes of solute transport along arteries both in the subarachnoid space and in the parenchyma. Extramural influx may occur (dashed green line) via a “periarterial space"between a sheath composed of pia and artery wall [11] or (solid green line) via the subpial space [129, 130]. Whatever their route, the solutes must cross the pia (green double-headed arrows) at some stage leading into the parenchyma. Efflux of solutes may occur by reversal of the extramural route (green dashed line) [12, 62, 96, 138] in which case, they would reach intramural sites within the smooth muscle layer by diffusion through the wall (double-headed red arrows in the main figure, dashed red lines in the insert). Alternatively [8, 129, 130, 139, 242], efflux may occur via an intramural route (solid red lines in the main figure and insert), which requires movement of solutes over long distances via the basement membranes of the smooth muscle layer of the arterial wall (light grey in the insert). For this route to be dominant there must be some feature of the arterial walls that prevents escape of solutes from the smooth muscle layer to the extramural periarterial space. The thick black lines represent the glia limitans at the surface of the brain parenchyma and surrounding the arteries. Structures shown in the figure are modified from those shown in Fig. 6 of [130]

The glymphatic hypothesis proposes that there is an extramural periarterial pathway (the dashed green line in Fig. 5) for influx, not efflux, and tacitly assumes that the net fluid flow carrying this influx is fast enough to exclude efflux by the same route. However, the presence of free-fluid filled extramural periarterial spaces within the parenchyma that could support a large inflow has been the subject of dispute.

- Weller, Carare, Morris, Abernathy and coworkers have put forward an opposing view that there are no such spaces, neither intramural nor extramural, and that within the parenchyma both periarterial influx and efflux occur via basement membranes [8, 10, 100, 129, 130, 145]. They base this view on the fact that they could not see free spaces in the fixed material they examined in their electron micrographs and confocal microscope images.
- However, others have observed spaces filled with dye in fluorescence images taken in vivo [11, 17, 45, 146]. Similar spaces were also detected in brain sections using fluorescent antibodies as the solutes [86].
- Furthermore, it is difficult to imagine how anything other than free fluid containing spaces could account for the rates of solute influx and for the large sizes of the solutes which can gain entry in vivo (see e.g. [70, 86, 130]).

It is of course conceivable, even expected, that free spaces would be dynamic, perhaps even to the extent of sometimes being patent, as seen by some, and sometimes not, as seen by others. Indeed as discussed in the next section changes in the dimensions of the spaces are central to most proposed mechanisms for the propulsion of fluid that is said to be involved in relatively rapid solute movements [83, 109–111, 138, 147–149].

Whether or not there are free spaces, Carare and associates [8, 129, 130] reasoned that influx and efflux rather than sharing a common route follow separate pathways: influx going by what can be called a sub-pial extramural pathway along the basement membranes of glial cells and pial cells (the solid green line in Fig. 5) and efflux following an intramural periarterial drainage pathway (IPAD) along basement membranes of the smooth muscle layer (the solid red line in Fig. 5).

Their results based on inspection of fixed brain sections demonstrated that, when injected into CSF, nanoparticles [130] or the fixable amyloid-β [129] were seen within 5 min along the pial and glial basement membranes, i.e. along an extramural pathway (the solid green line in Fig. 5). After 30 min the amyloid-β was found in the smooth muscle layer of the arteries. By contrast when injected into the parenchyma similar solutes (dextrans, biotinylated amyloid-β and a fixable fluorescent derivative of amyloid-β) were seen at sites in the basement membranes of the smooth muscle layer of arteries but not, except at the site of injection, along the extramural route. They interpreted these results as evidence for an intramural efflux pathway leading outwards from the parenchyma. Provided escape from the intramural pathway across the vessel wall (see Fig. 5) is sufficiently slow, such a pathway could explain how large hydrophilic solutes are delivered to the ipsilateral arteries connecting to the circle of Willis [96, 138] (see preceding section).

However, it should be noted that such appearance of solutes in arterial walls far removed from the parenchyma remains the only clear evidence for solute movements occurring along intramural pathways [62, 96, 138]. The more recent evidence is consistent with efflux occurring either via the intramural route or via the extramural route shown in Fig. 5 in the first case by long distance intramural movement within the smooth muscle layer as proposed by Carare and colleagues and in the second case by long distance extramural movement combined with penetration into the arteriolar or arterial wall as proposed to occur in the spinal cord by Liu and colleagues [143]). It should be born in mind that there may be changes occurring in the tissue after death but before fixation is complete, which might putatively collapse the extramural periarterial space displacing solutes into the basement membranes around the smooth-muscle cells and so accounting for the intramural location seen using fixed material [45].

Evidence in favour of an extramural periarterial efflux pathway has been presented by Arbel-Ornath et al. [12]. Using in vivo two-photon imaging of 3 kDa cascade blue dextran they found that after pressure injection of the dye into cortex the dye rapidly accumulated along arterial walls (not veins). Later most of the dye was still detected extramurally though some of it became evident in the smooth muscle layer. Arbel-Ornath et al’s results echo the earlier results of Szentistvanyi et al. [62] who reported that horseradish peroxidase injected into the midbrain could be found 70-120 min later concentrated in the “periadventitial tunic or perivascular sleeve” of the basilar and “pericerebral” arteries, but not the " thick muscular tunic of these vessels “.

#### The driving forces for periarterial influx and efflux of solutes

The driving forces for periarterial fluxes have not been identified with any certainty. The first theory was that of Bradbury, Cserr and Westrop [138] who proposed that expansion of penetrating arteries during systole would expel from the parenchyma the contents of the periarterial spaces while in diastole relaxation would pull the fluid back in. While this idea sought to explain how efflux of solutes from the parenchyma might take place, it would also account for influx with the net flux determined by the difference in concentrations in the parenchyma and in the sub-arachnoid space. However, as discussed below, it is now thought that arterial pulsations tied to the heartbeat are too small for this mechanism to occur but the idea may warrant reconsideration in connection with slower changes, perhaps driven by respiration [150–154], by changes in CSF flow [155] or by vasomotion (or vasomotor waves) [31, 149, 156, 157].

Expansion and contraction of arteries during the cardiac cycle do occur and it is plausible that these could bring about changes in periarterial spaces. In each human cardiac cycle about 0.6 mL of CSF shifts from cranium to spinal cord and back as a result of the cyclic increase and decrease in volume of the vasculature [158].^13^ The vasculature is presumed to expand and contract by a similar amount, which is about 1% of the total vascular volume in the brain. Iliff et al. observed similar changes in penetrating vessels of mice. They argued that rather than the periodic filling and emptying previously envisaged these pulsations drive inward periarterial flow by peristalsis [11, 83]. Further support for this explanation has been obtained from the reduction in periarterial entry of markers caused by hypercapnia. Hypercapnia causes arterial dilation which if maximal would both reduce the size of the periarterial spaces, increasing the resistance to inflow, and reduce the changes in size during the cardiac cycle thus eliminating the proposed driving force for inflow [159]. The suggestion of periarterial peristalsis could, of course, only account for the influx of solutes.

There are at least four arguments that inward flow carrying solutes is not the entire explanation of periarterial fluxes.

- There is likely to be periarterial efflux of solutes. If the efflux occurs via the same pathway as the influx, then the process isn’t simply carriage of solutes by flow. Alternatively if the efflux occurs by a separate but still periarterial route, then that route must be taken into account explicitly.
- There are solutes, e.g. India ink particles and possibly high molecular weight dextrans, that can penetrate into parenchymal periarterial spaces but cannot enter the parenchyma. If such a solute is present in the subarachnoid space for long enough and is swept into the periarterial space, then either it must fill and plug the space or there must be some way for it to get out, most plausibly by traversing in reverse the pathway by which it got in. Brierley [70] observed entry of India ink particles over at least 24 h and found levels in the spaces at all depths similar to those in the adjacent region of the subarachnoid space. Iliff et al. [11] only followed entry of high molecular weight dextran for 25 min which unfortunately was insufficient to tell if the dextran concentration approached a value less than, similar to, or greater than that in the surface periarterial space. More informative is Brierley’s result that suggests India ink can get into and out of the extramural periarterial spaces at similar rates and thus that solute transport there is bidirectional.
- AQP4 knockout (see Sect. 5.3) has been reported roughly to halve solute transfer from cisterna magna to parenchyma (see Fig. 4F in [11] but see also Fig. 4I which suggests a much larger decrease). In the glymphatic hypothesis this is interpreted as meaning that knockout reduces the rate of circulation, i.e. that it reduces the rate of inflow along the periarterial routes and the rate of entry into the parenchyma. However, in the same study the rate of entry of a large dextran into the periarterial spaces was not significantly reduced which suggests either that AQP4 knockout does not change the flow in periarterial spaces (see Smith et al. [120]) or that flow is not the mechanism leading to movement of the solutes within the periarterial spaces.
- Theoretical studies have investigated whether small changes in diameter of penetrating arteries could provide a net periarterial flow adequate to account for experimentally observed influx or efflux. The initial studies suggested they could [160, 161] but subsequent studies have generally concluded that they cannot [148, 162]. It also looks likely that Kedarasetti et al’s [108] argument that peristalsis along subarachnoid periarterial spaces would require much larger changes in the space width than observed applies as well to parenchymal periarterial spaces.^14^ Much of the theoretical work on flow in these spaces has been reviewed by Thomas [109], Martinac and Bilson [110] and Faghih and Sharp [111]. Thomas comes to somewhat different conclusions than Kedarasetti et al. It should be noted that some studies relate primarily to subarachnoid periarterial spaces and others to parenchymal periarterial spaces. All efforts to compare model predictions with data for perivascular transport *within the parenchyma* are limited by the paucity of experimental data for the dimensions of the spaces and how these vary, and the impossibility (at least at present) of seeing the movements of solutes actually within the spaces.

These observations cast serious doubt on the idea that solutes are transported simply by being entrained in a net flow along an extramural periarterial space but they do not provide any alternative mechanism. Asgari et al. [148] favour dispersion or mixing caused by oscillatory flow which could in principle account for influx or efflux depending on the concentration gradient of the solute. However, Sharp et al. [163] and Troyetsky et al. [164] conclude that such mixing would produce very little net flux of solute.

Separation of the influx and efflux routes for solutes would allow the possibility that influx is tied to inflow of CSF while efflux is not. In a theoretical study Diem et al. [162] concluded that arterial pulsation could not drive the observed efflux. Aldea et al. [149] reinforced that conclusion and suggested that the only mechanism that could explain efflux via the intramural route is vasomotion, i.e. contractions of the smooth muscle cells. In support of this idea, vasomotion in surface arterioles in the visual cortex has been visualized in vivo, and shown to be modulated by visual stimulation [157]. In addition the extent of vasomotion appeared to correlate with the rate of clearance of the particular markers used. Further study is required [165].

In summary it is still not understood what forces drive periarterial fluxes of solutes or the exact routes that they take. If, as is strongly supported by the existing data, there are solute fluxes both into and out of the parenchyma, the mechanism(s) of periarterial transport cannot be described simply as an inflow of CSF.

#### The fraction of CSF that enters the parenchyma via periarterial routes

The glymphatic hypothesis tacitly assumed that a large fraction of the CSF produced would enter the parenchyma via the periarterial spaces.

The average inward net flow along periarterial spaces is still unknown. Even the proportion of injected markers reaching the parenchyma from the subarachnoid space is not known with any certainty. Efforts have been made to obtain data which allow the distribution of markers and their uptake into the parenchyma to be determined quantitatively. In one of the first attempts, Papisov et al. [134] found that “at 2.5 h after the injection up to 15% of the intrathecally administered dose of proteins and phage particles can be localized in the brain volume (excluding the ventricles)”. However, most of those large markers that did reach the brain may have remained in CSF-containing spaces rather than entering the parenchyma itself. More recently, Lee et al. [118] endeavoured to determine the amount of a much smaller marker, Gd-DOTA, within the brain (excluding the large CSF spaces) of a rat anaesthetized with dexmedetomidine/isoflurane (chosen to maximize inflow) after addition of a known amount at the cisterna magna. They found that about 20% of the Gd-DOTA entered the parenchyma, the rest being eliminated without entering. Thus concentrations within the parenchyma were initially well below those in CSF (in the cisterns). The time course showed that the amount within the brain (excluding the large CSF spaces) was maximal about an hour after addition to the cisterna magna but subsequent elimination extended over a much longer time with the concentration within the parenchyma falling by only about 20% in the next hour. The data reported are consistent with there being influx into the parenchyma while CSF concentrations are greater than parenchymal concentrations but relatively quickly reversing to efflux when the CSF concentrations are reduced by elimination. The decline in total amount present in the brain (excluding the large CSF spaces) would then represent a slow efflux consistent with the idea that only a small percentage of administered Gd-DOTA enters the parenchyma.

**Fig. 6:**
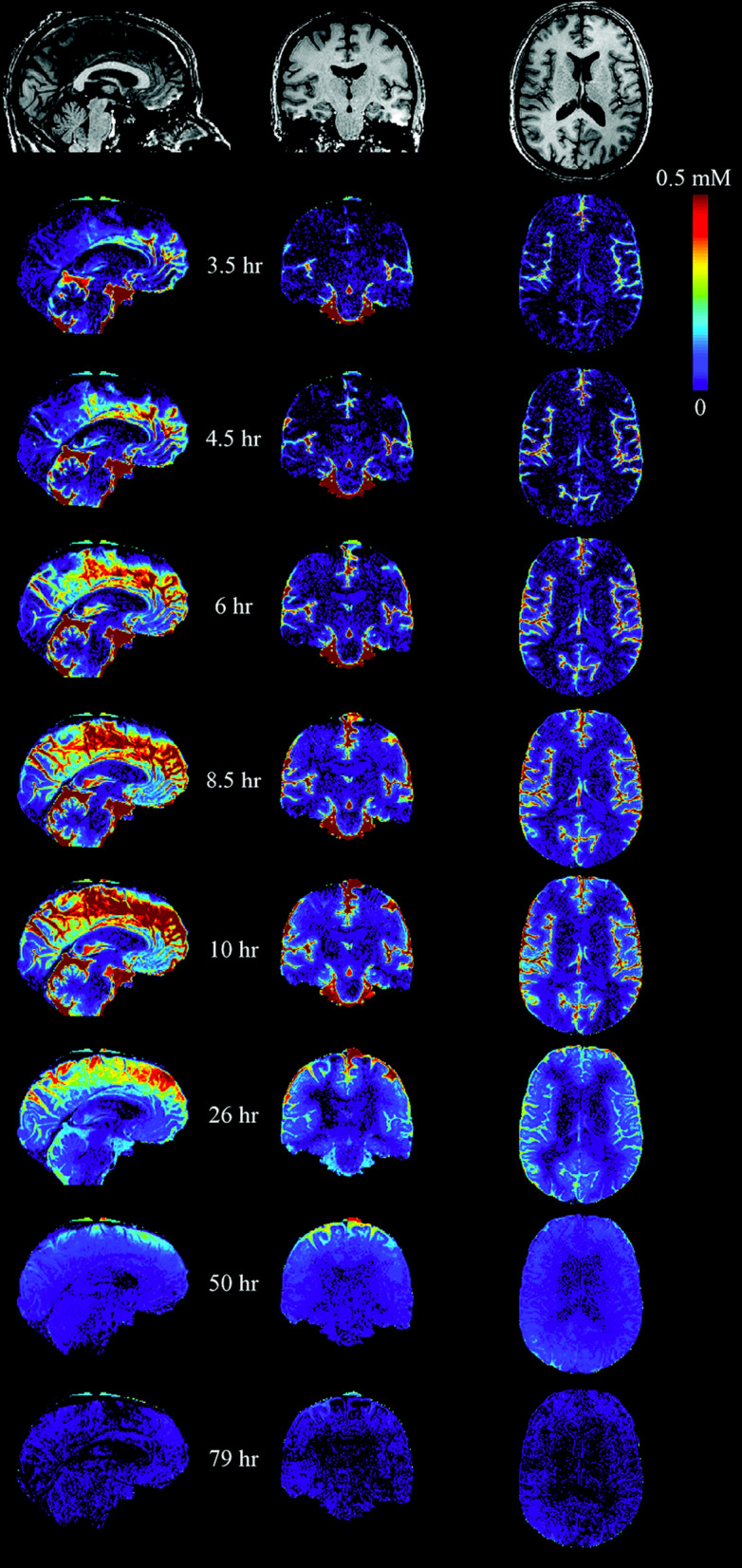
Human MRI images showing changes in gadobutrol concentration following an intrathecal injection. At *t *= 0, 1 µmol of gadobutrol was injected into the subarachnoid space of the spinal cord. It reached the cisterna magna in about 3 h and spread over the surface of the brain in the next 7-8 h. Note that the gadobutrol concentrations on the dorsal surface of the brain persist for longer than in the basal cisterns as expected if elimination of gadobutrol occurs primarily from the cisterns, e.g. across the cribriform plate, but not from the dorsal subarachnoid spaces. The partial analysis of these data in [117] shows that in various regions of the brain the concentrations in grey matter are still increasing up to 12 h and subsequently decrease over days in parallel with that in CSF adjacent to the region. However, even at their maximum they are less (2 to 5 fold depending on region) than the concentrations that were achieved in the adjacent subarachnoid spaces. At the very latest times, the concentrations in parenchyma appear to exceed the then current concentrations in adjacent subarachnoid spaces. The subject was awake from the time of administration until after the scan at 10 h and had a normal nights sleep before each of the last three scans. Figure taken with permission from Watts et al., Am. J. Neuroradiol., 2019 [117]

Watts et al. [117] obtained data from a human using an MRI extracellular fluid marker, the contrast agent gadobutrol, but as of the time of this review a kinetic analysis of these data has not been published. Bearing in mind the previous results with rodents, the most remarkable feature of the results for the single human subject in Fig. 6 is the long time scale, which echoes an earlier result of Eide et al. [166]. However, without detailed analysis it is difficult to establish with any confidence whether the slow steps represent the distribution and outflow of CSF or the entry into and exit from the parenchyma of the contrast agent. The persistence of the agent in the parenchyma whilst that in the CSF had been reduced suggests that efflux from the parenchyma and hence also influx were slow. This argues that the proportion of gadobutrol crossing into the parenchyma was low.^15^ A full study with analysis of data from more than one experimental subject would be very informative.

Bearing in mind that any values for the flow into the parenchyma inferred from the experiments of Lee et al. and Watts et al. are derived from measurements of the entry of markers, the flows inferred could well be overestimates if marker entry is facilitated by convective mixing in the periarterial spaces.^16^

To re-iterate the main points of Sects. 5.2 to 5.2.5: The data show that there can be both influx and efflux of solutes by periarterial routes but do not support the idea that there is entry into the parenchyma of a large fraction of markers added to CSF. The data have not established the rate or extent of fluid entry into the parenchyma by periarterial routes. On the other hand it should be noted that it has proven difficult to provide convincing arguments for any mechanism other than net fluid flow to account for the inward movement of solutes faster than would be possible by diffusion alone.

### Fluxes and flows across endfoot layers. The role of AQP4

In the original papers proposing the glymphatic hypothesis AQP4-knockout was reported to have effects on solute movements, decreasing their influx from the cisterna magna to the cortical parenchyma and their elimination from the parenchyma [11]. The hypothesis proposed that AQP4-knockout did this by decreasing the water permeability of endfoot membranes which in turn decreased the flow of fluid across the endfoot layers and hence the glymphatic circulation. This was an obvious idea to consider because AQP4 channels in the endfoot membrane had been shown to be important in influencing osmotically driven water influx from blood to astrocytes [167, 168]. However, as early as 2015 it was pointed out by Smith, Jin and Verkman [169] that water flow through channels in the blood vessel facing membranes of glial endfeet is unlikely to mediate hydrostatic pressure driven fluid transfer between perivascular space and interstitium.

There are a number of arguments in support of the contention that the water permeability produced by AQP4 in the endfoot membrane is not necessary to provide a route for water transfer between the periarterial space and the interstitium under physiological conditions:

- The endfoot layer is not a tight layer and so water and solutes could easily flow via the gaps between the endfeet. Even the smallest estimates of gap sizes [170] would still be large enough to allow water flow to be primarily via the gaps (see Fig. 5 in [171]). For recent calculations of flows based on this premise see [172]).
- Whether or not AQP4 is present in the thin endfoot layer the water permeability of that layer with its cells and gaps is still higher than that of the much thicker, adjacent region of interstitium. Hence the limitations on fluid flow reside primarily in the adjacent interstitium and not in the thin endfoot layer [171].
- The presence of AQP4 in the endfoot membrane facing the endothelial cells increases the water permeability by only about 2.5-fold [173].^17^ (This increase is temperature dependent, the figure quoted here is for 37 °C.)
- AQP4 is not itself permeable to solutes and it is the movement of a solute, NaCl, that is likely to be the rate limiting step in any flow of fluid driven by a hydrostatic pressure difference. Water moving without solute would create local solute concentration changes which, though small, would still produce an opposing osmotic gradient sufficient to limit water movement [3, 39, 169, 174–176].^18^, ^19^
- Smith et al. [169] argue that anything that diverts water flow from the gaps to a trans-endfoot route would be expected to reduce solute transport through the gaps by fluid flow. *But* the reported effect is greater solute transport when AQP4 is present than when it is absent [11, 122]. The inference they draw is that however AQP4 increases solute movements it may be doing so in a less direct way than increasing trans-endfoot water movement.

A glymphatic circulation requires that both water and solutes cross the endfoot layer. Whether or not AQP4 is present the water can cross. However, the routes and driving forces for solute movements across the layer have not been explained.

There is evidence that net transfers of solutes in and out of the parenchyma are influenced by the presence of AQP4. Thus:

- the rate of solute transfer from cisterna magna into the parenchyma is reduced in AQP4 knockout mice [11, 122] (but see below);
- expression levels of AQP4 are correlated with changes in rates of perivascular influx and efflux of markers (see e.g. [122, 177, 178];
- AQP4 knockout in mice reduces both the development of oedema following middle cerebral artery occlusion [167] and the rate of resolution of vasogenic oedema [179]. The latter and to some extent the former^20^ depend upon movements of solutes, primarily NaCl;
- inhibition of AQP4 reduces transfer of Gd-DOTA and Gd-DTPA (low molecular weight extracellular fluid markers that can be followed using MRI) from CSF into the parenchyma [180, 181] and reduces the clearance of tau [181].

The original observations of the effects of AQP4 knockout have been refuted by Smith et al. [182] who asserted that the knockout does not in fact decrease the influx of solutes from the cisterna magna to the cortical parenchyma. In response, Mestre et al. [122] maintained that the opposite was true, i.e. that knock-out does decrease solute influx, at least in the hands of four different laboratories. They go on to point out that the choice of avertin (tribromoethanol) as anaesthetic by Smith et al. was very unfortunate in that it appears to suppress the transfers from cisterna magna to interstitium and assert that this explains their negative results. In reply, Smith et al. [120, 183] point out that the effects reported by the four laboratories are all much smaller than those described in the earlier publications (see also Sects. 5.2.1 and 5.2.5). Using ketamine/xylazine anaesthesia (as in the studies reporting that knockout does have effects) together with a high rate infusion into the cisterna magna to achieve reproducible delivery to the cortical surface, Smith et al. [120] confirmed their earlier result that penetration of the dextrans was the same in wildtype and AQP4 knockout mice. Furthermore using direct application of markers to the cortical surface combined with imposing a small constant hydrostatic pressure, they found that the knockout had no effect on the easily measured penetration of the dextrans into periarterial spaces and into the parenchyma across the glia limitans at the cortical surface. However, they acknowledged that the last of these results did not exclude the possibility that AQP4 might affect solute transfers across the glial endfoot lining of the perivascular spaces.

The balance of available evidence is that there are effects of AQP4-knockout on transfers of markers from remote sites of administration into the parenchyma. However, the reasons why these effects occur are not clear (for discussion of these issues from a different vantage point see [34]). In reinterpreting the data it will be important to remember that changes in the measured rates of transfer of solutes from cisterna magna to the parenchyma may in some circumstances result from changes in CSF flow to the regions being inspected [53, 120] rather than in their transfer from CSF into the parenchyma. Changes in CSF flow can be very important (see Sect. 5.1, [37], Fig. 5 in [177, 38], Sect. 2.4 in [18, 121] and [120]).

It has been suggested that one way that AQP4 knockout could affect fluid and solute movement is by altering swelling of the endfeet [168, 169, 172]. For instance, Amiry-Moghaddam et al. [168] suggested that knock-out of AQP4 from the endfoot membrane, would have the effect of reducing outflow of metabolically-produced water from the endfeet hence leading to their swelling. Such swelling might decrease the width of the gaps between the endfeet and thus increase resistance to fluid movement from the periarterial spaces into the interstitium. There may, of course, be other changes. However, it should be noted that there is at present no compelling evidence for any particular mechanism for the effect of AQP4 on solute transfers.

Studies on efflux from the parenchyma might be easier to interpret than the studies on influx discussed above. How AQP4 affects perivascular flow and transport of solutes into and out of the brain is far from settled and clearly requires further investigation (see [184], Sect. 4.3.3 in [3, 5], the contributions to a Crosstalk debate in the Journal of Physiology [48, 45], and [34]).

### Fluxes and flow through the interstitial spaces of the parenchyma

There is no published experimental evidence that demonstrates fluid flow within the interstitial spaces of grey matter undisturbed by oedema or ongoing infusion.^21^ The classical work by Cserr and colleagues on the extravascular clearance of solutes from the parenchyma was interpreted in terms of there being diffusion in the interstitial spaces and flow along “preferred routes”. On present evidence there is no reason to depart from their view. Sophisticated analyses of the data from MRI experiments with gadolinium probes [103, 123, 124] have reinforced the belief that there is entry of solutes from CSF into the parenchyma (see Sect. 5.2) and that diffusion alone cannot account for all of this transport. However these studies have not established that there is flow within the interstitium in addition to that in the perivascular spaces and other “preferred routes” (see Footnote 22).^22^

By contrast to the lack of evidence for flow in the interstitial spaces, there is abundant evidence for diffusion. This has been obtained primarily using real time ionotophoresis and/or the spreading of fluorescence immediately following injection of fluorescent dyes (see [185] for a review). In addition, Smith et al. [182] found that diffusion accounts entirely for recovery of fluorescence after photobleaching of a previously injected dye.

Countering Smith et al’s argument that diffusion is sufficient, Mestre et al. [122] asserted that any acute procedure involving injection of a solute into the parenchyma would lead to a global suppression of glymphatic flow^23^ (see also [186–188]) leaving only diffusion to account for the movements of solutes. If such a suppression were indeed to occur and be both sufficiently complete and sufficiently long lasting, this would invalidate the interpretation of almost every experiment that has sought to investigate flow within the parenchyma including those interpreted as supporting the glymphatic hypothesis. It is not yet clear that the suppression is either complete (see footnote 23) or long-lasting. These are important issues that require further investigation.

Irrespective of Mestre et al’s objections, there are still at least two compelling arguments against an important role for flow in solute movements within the interstitium. The first is that the pressure differences needed would exceed any that are possible. The second is that the flow required would far exceed that which could be provided by CSF flow.

**Fig. 7:**
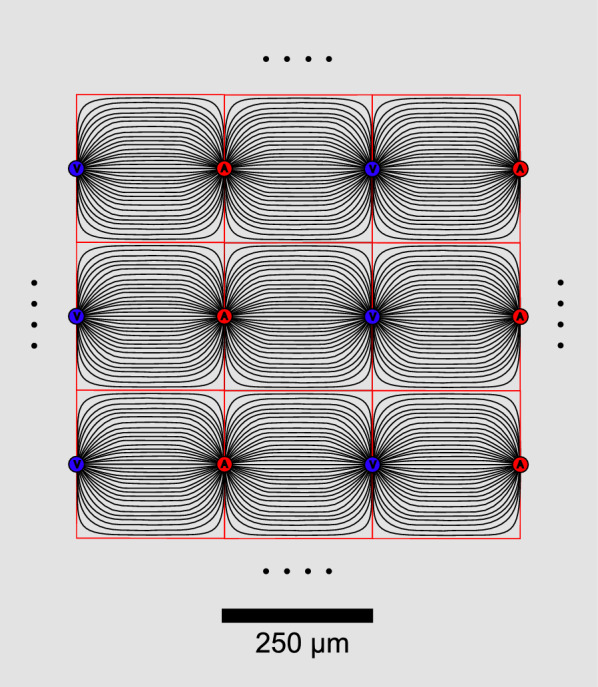
Schematic diagram of a cross section of the theoretical array of parenchymal blood vessels in the model used by Ray et al. [193]. In this model the vessel array is approximated by a regular repeating pattern of arterioles (red circles) and venules (blue circles) running perpendicular to the cortical surface. Streamlines (thin black lines) connect arterioles the sources of flow, and venules,- the sinks. Conveniently for the calculation of flow from the flow velocity the midplanes between the planes of arterioles and venules separate the sources from the sinks and the direction of flow is perpendicular to the midplanes (see footnote 26). Note that the cross-sectional area available for perivascular flow along the arterioles and venules is much smaller than the area available for flow through the interstitium. Thus, with the same flow inwards along periarterial spaces, through the interstitium and outwards along perivenous spaces, the flow velocity would be much higher in the perivascular spaces than in the interstitium

- Theoretical calculations have concluded that the narrowness of the interstitial spaces in grey matter means that the resistance to flow is so large that flows produced by achievable hydrostatic pressure differences will be small and thus will have insignificant effects on solute movements compared to diffusion^24^ [39, 171, 182, 189–192].
- The second argument is based on an estimate of the flow through the parenchyma that would be required to alter the symmetrical spread of solutes resulting from diffusion, i.e. the flow sufficient to deliver solutes to venules rather than to arterioles. Ray et al. [193] thought that asymmetry induced by flow could explain the scatter of results seen in real-time iontophoresis experiments. To be able to make their calculations, they used a simplified model of the distribution of blood vessels in the brain parenchyma (see Fig. 7) and concluded that the velocity^25^ of the flow midway between arterioles and venules would have to average more than 10 μm min^−1^ for flow to produce observable asymmetry [193, 194]. This value is consistent with previous comparisons of the relative importance of flow and diffusion based on the Peclet number [185, 195].

Ray et al’s model provides a method to calculate either the flow velocity or the flow in the parenchyma if the other is known.^26^ Using their model it follows that the total flow required in a rat for the flow velocity to be as important as diffusion in the movement of solutes would be more than 40 times greater than the total CSF production rate and even more so in humans. This value challenges the original idea that flow is sufficiently large as to be able to eliminate extracellular solutes from the interstitium. Sweeping wastes out of the parenchyma would require flows so large that CSF would have to be recirculated through the parenchyma many times before it leaves the brain, but the available evidence (see Sect. 5.2.5) suggests that the flow entering the parenchyma via periarterial routes is substantially less than the CSF production rate.

It is apparently now accepted by all that diffusion plays a significant role in solute transfers in the interstitium (see e.g. [45, 194]). Indeed, most investigators [5, 171, 182, 183, 185, 191, 192, 195] have concluded that diffusion is almost certainly the dominant means for delivering solutes *rapidly* over the *short* distances from interstitium of grey matter to brain surfaces, perivascular spaces and white matter.^27^ There is no evidence for flow sweeping solutes towards perivenous spaces (as portrayed in Fig. 1) or indeed towards any other “preferred routes” of outflow.

### Extravascular efflux of solutes from the parenchyma

Periarterial efflux of solutes was considered in Sect. 5.2. Evidence for other efflux routes is discussed in the following two sections.

#### There is little evidence for solute efflux or fluid flow from the parenchyma along perivenous routes

The glymphatic hypothesis proposed that there is a fluid flow that sweeps solutes through and then out of the parenchyma and thence via perivenous pathways outwards to either CSF or lymph. All evidence is now against the idea that the solutes flow through the parenchyma and then into nearby perivenous spaces or indeed into any other spaces. However, it seems likely that there is some form of net flow or mixing within “preferred routes” for efflux that is important in moving solutes the relatively long distances to the cortical surfaces (see Sects. 2.1 and 5.4 and compare [28, 109]). By reducing the concentrations of wastes along routes for efflux, efficient movement along “preferred routes” would produce a concentration gradient for diffusion of the wastes out of the parenchyma to those routes just as in the classical hypothesis (compare [109]).

A net perivenous fluid outflow, such as required by the original glymphatic hypothesis, may exist but no one has found a way to see it (see e.g. [126]). Rennels et al. [75] using horseradish peroxidase injected into the ventricles and Iliff et al. [11, 15] using ovalbumin injected into the cisterna magna reported evidence that markers do reach the walls of veins draining the cortex somewhat later than they reach cortical periarterial spaces. Indeed this was a major part of the evidence advanced in favour of there being a circulation of fluid delivering solutes: firstly to periarterial spaces of surface vessels in the subarachnoid region, then to periarterial spaces next to vessels within the parenchyma, then to parenchymal tissue spaces and finally to certain large draining veins [11, 196]. However, there is no evidence that markers follow a perivenous route within the parenchyma to reach these large veins.

There is only limited evidence either for or against influx or efflux of solutes via specifically perivenous routes [11, 34, 47, 64, 75, 84, 196, 197].^28^ More recently Rasmussen et al. [34] discuss several extravascular efflux routes for solutes from the parenchyma including transfer from the parenchyma to the walls of large veins by routes that do not trace the intermediate portions of the venous vasculature. Alternatively late appearance around veins of markers added to CSF may reflect slow arrival directly from CSF rather than entry to the parenchyma and subsequent efflux [148]. Favouring direct access of markers from CSF into perivenous spaces, Jolly et al. [198] reported that N-sulphoglucosamine sulphohydrolase given intracisternally penetrated periarterial and perivenous spaces and similarly Pizzo et al. [86] reported that antibodies given intrathecally reach the parenchyma via all perivascular spaces including those of veins.

#### Is there a fluid outflow that can account for solute efflux from the parenchyma?

The contention that flow has no observable effects on solute movements within the interstitial spaces does not imply that flow cannot still be important in the perivascular spaces within the parenchyma. One way in which such flow could be sustained has been suggested by Abbott, Pizzo, Thorne and colleagues [5, 86, 127]. They have proposed that periarterial flow can continue into a pericapillary space from which it can then provide a perivenous flow. However, unless there is a gap containing free fluid between the basement membranes of the endothelial cells and the glial endfeet, as is suggested by Abbott et al., the resistance to flow along the pericapillary sleeve is likely to be too high [195]. Alternatively a pericapillary route may not be needed. As discussed below a flow sufficient to account for elimination of solutes by preferred extravascular routes may still be too small to affect the diffusion of solutes in the interstitium.

It remains to be considered whether a major portion of solute efflux from the parenchyma can be accounted for theoretically by a flow inwards along extramural periarterial spaces and outwards via perivenous spaces and, probably more importantly, other “preferred routes” of efflux. The flow required to account for such solute efflux can be estimated from the clearances of solutes that are eliminated by extravascular transport. In rats these are about 1 µL min^−1^ g^−1^ (see Sect. 3.2 and Table 1 in [2]). A flow of 1 µL min^−1^ g^−1^ in grey matter would produce a superficial flow velocity of only ~ 0.25 μm min^-1^ in the interstitium [199] (see footnotes 26 and 27) which would produce negligible effects there on solute movements compared to diffusion. By contrast when considering efflux via the preferred routes, the distance to be covered is much larger and the cross-sectional area available for flow/diffusion is much smaller which may mean that flow there is dominant.

In rats a flow of 1 µL min^−1^ g^−1^ would require the flow into, through and out of the parenchyma to be as high as the rate of CSF production in the ventricles.^29^ The results of Lee et al. [118] showing that only about 20% of CSF enters the parenchyma in anaesthetized rats and of Ma et al. [53, 200] indicating that at least under some circumstances at most a very small percentage enters the parenchyma are obviously incompatible with the idea that elimination from the parenchyma is primarily by such a circulation of CSF flowing into the parenchyma and subsequently out again. It follows either that net fluid outflow is not the mechanism for elimination of solutes from the parenchyma or that there is some additional source of fluid, e.g. inflow of fluid across the blood–brain barrier. The qualitative conclusions are the same for humans (see footnote 29).

The rate of interstitial fluid (ISF) secretion across the blood–brain barrier is still unknown; it could be negligible or even larger than the rate of CSF production. The balance of evidence suggests that there is some secretion into the parenchyma [1]. The principal argument against a substantial rate of ISF secretion is that the rate of CSF secretion assessed by ventricular-cisternal perfusion is similar to the total rate of fluid secretion into the brain assessed by lumbar drainage (see footnote 10 in [1]). However, those rates might still be similar in the face of a substantial rate of ISF secretion into the parenchyma if ISF were to leave the parenchyma by extravascular transport to lymphatics (see next section) or by any other route whose rate was independent of whether or not there was lumbar drainage of CSF.

In summary, at present it remains possible that extravascular solute efflux from the parenchyma might occur by means of fluid outflow as proposed in both the classical and glymphatic hypotheses. If so, a source of fluid other than just periarterial inflow is required. This may be secretion of ISF across the blood–brain barrier. However, it should be noted that the alternative, that efflux takes place by other forms of convection, e.g. mixing, is also possible.

Perhaps the most commonly used type of argument in favour of the glymphatic hypothesis is that a factor altering extravascular entry of solutes also alters extravascular efflux of solutes as if both occur via a change in the rate of fluid circulation. These factors include: knockout of AQP4 [11]; sleep [37]; anaesthesia (especially that incorporating the α_2_ adrenoceptor agonists xylazine or medetomidine) [37, 201]; traumatic brain injury [15]; age [177]; posture [202]; ablation of meningeal lymphatics [203]; small vessel disease [19]; idiopathic normal pressure hydrocephalus (iNPH) [116]; stroke [14]; and hypercapnia [159]. However, these qualitative comparisons do not prove that a circulation of fluid through the parenchyma exists. For example, similar results might be obtained if solute entry from the cortical surface and solute efflux from the parenchyma were both periarterial, i.e. even in the complete absence of a parenchymal circulation (see Sect. 5.2).

### Do solutes and fluid emerging from the parenchyma directly enter lymphatics?

**Fig. 8:**
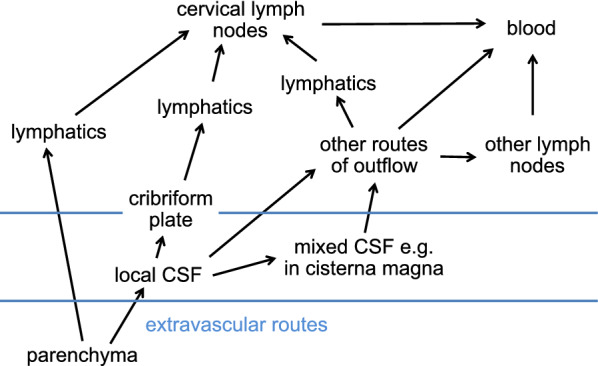
Flow diagram of routes taken by large hydrophilic solutes emerging from the parenchyma by extravascular routes. The eventual destination is blood but there are multiple routes that a solute can follow. The initial extravascular pathways be they periarterial, perivenous, subependymal or white matter tracts may lead to CSF. In addition, particularly the intramural perivascular pathways may lead to lymphatics. Solutes that reach CSF at the parenchymal surface in anterior and/or ventral portions of the brain may be taken via the olfactory nerve to the cribriform plate and thus to lymphatics. Alternatively the solutes may be mixed into CSF that at some point traverses the cisterna magna, perhaps on route to spinal sites of elimination. A significant portion of the CSF may also be directed to lymph. It cannot be assumed that solutes reaching the cervical lymph nodes have not first mixed with CSF

CSF is an obvious destination for solutes and fluid emerging from the parenchyma by “preferred routes” be they white matter tracts, subependymal spaces or perivascular spaces. However, there is an important alternative in that efflux and outflow via perivascular pathways may be directed to lymphatics without first mixing with CSF (see Fig. 8) [53, 63, 66, 67, 94, 159, 204–208] and for a more sceptical view [209]. Rasmussen et al. [34] have proposed that as much as 80 % of ISF leaves the brain directly to lymph without mixing with CSF in the subarachnoid spaces or ventricles.^30^ This is an attractive suggestion because it would mean that wastes emerging from the parenchyma by extravascular routes would not enter CSF and possibly be recirculated into the parenchyma. (Note this would also exclude recirculation of CSF as a mechanism to increase flow through the parenchyma.) The glymphatic hypothesis is consistent with efflux and outflow going to either CSF or lymphatics.

For large solutes there is good evidence for efflux from the parenchyma to meningeal lymphatics either running parallel to the venous sinuses or at the base of the skull [90, 95, 206, 207, 209–211]). In the original glymphatic hypothesis it is proposed that efflux occurs by perivenous outflow. This can easily be modified to include other efflux routes, e.g. white matter tracts and subependymal spaces. As noted in Sect. 5.5.2, to account for efflux the outflow must be similar to the entire rate of production of CSF all of which would have to be directed into the parenchyma. If 80% of the CSF flowing into the parenchyma goes out directly to lymphatics rather than returning to CSF then 80% of the CSF produced would leave the brain via this route and very little would be available to flow out by other routes. However, there are major outflows of CSF via the cribriform plate and spine [209]. Furthermore, it has been shown that absence of meningeal lymphatics does not alter intracerebral pressure or cerebral water content which argues against these lymphatics being a major route for outflow of fluid [206, 211]. If they were the principal means for fluid outflow, then their absence would have substantially increased pressure, much as was seen when another proposed route of outflow, that across the cribriform plate was blocked [212, 213]. At present there are no compelling arguments either for or against there being a large outflow direct to lymphatics.^31^

The role of lymphatics in the CNS has been reviewed recently by Da Mesquita et al. [90], who unfortunately ignored other possible routes by which solutes could reach lymph from CSF. By contrast Frederick and Louveau [211] in their more recent review on lymphatics did consider alternative routes. The present position appears to be that a substantial percentage of the efflux of large hydrophilic solutes from the parenchyma is directed to lymphatics but, as inferred from the measured effects on pressure, only a small percentage of the outflow of fluid takes such a route. The mechanism for the separation of the large solutes from the fluid flow needs investigation. In this regard it is important to note that the meningeal lymphatics are in the dura and are thus separated from the CSF by the arachnoid barrier. For further discussion of the role of meningeal lymphatics in efflux see [209].

## Summary

The glymphatic hypothesis in its original form proposed that a flow of fluid circulating into, through and out of the parenchyma could account for the influx and efflux of those solutes that cannot move across the blood–brain barrier and so depend upon extravascular routes. Perhaps the most commonly used argument in favour of the hypothesis has been that many factors that increase or decrease influx also affect efflux in the same way. These factors include knockout of AQP4, sleep, anaesthesia incorporating the α_2_ adrenoceptor agonists xylazine or medetomidine, age, posture, small vessel disease, idiopathic normal pressure hydrocephalus (iNPH), stroke, and hypercapnia (see Sect. 5.5.2).

Unfortunately, much of the available evidence raises doubts about the validity of the glymphatic hypothesis at least in its original form. Here are some of the confounding factors.

- The resistance to fluid flow in the interstitial spaces is so large that flows produced by achievable hydrostatic pressure differences will have insignificant effects on solute movements compared to diffusion (Sect. 5.4).
- The flow that would be required to sweep solutes out of the parenchyma into perivenous spaces or into other “preferred routes” far exceeds any flow that could be supplied by CSF flow (Sect. 5.4). Solute movement within the interstitial spaces of grey matter in the parenchyma is by diffusion and except in oedema or during infusions of fluid is not driven by fluid flow.
- Both influx and efflux of solutes can occur along the periarterial pathways (Sect. 5.2). Thus transport along these routes cannot be just an inward flow.
- The evidence that efflux of solutes from the parenchyma is perivenous comes solely from studies showing that markers entering the parenchyma from CSF or present in the parenchyma following injection can be found subsequently along the walls of large veins (Sect. 3). However, it cannot be concluded from these observations that the solutes are leaving the parenchyma by perivenous routes because they may have gained access to the vessel walls by alternative routes (Sect. 5.5.1).
- The glymphatic hypothesis proposes that solutes leave the parenchyma carried by fluid flow. The minimum putative outflow required is the solute clearance of suitable markers (see Sect. 5.6) which can be measured. However, this calculated outflow in all cases for which clearances are available is as large as the entire measured rate of CSF production in the ventricles. But in a glymphatic circulation periarterial inflow must equal outflow and thus the inflow must also be as large as the CSF production rate. This is not seen. Current estimates of inflow are less than (perhaps much less than) 20% of CSF production rate.

**Fig. 9:**
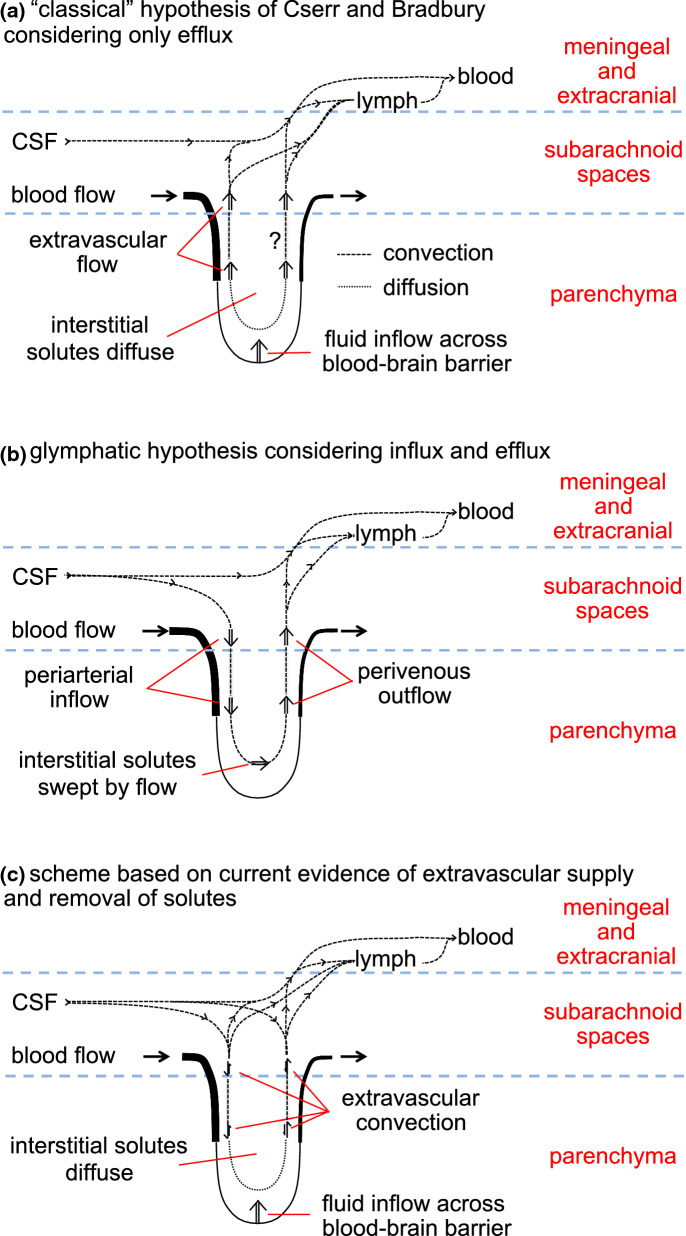
Comparisons of the classical hypothesis, the glymphatic hypothesis and a scheme based on current evidence. They each summarise processes that may be important in extravascular supply and removal of solutes. **a** In the classical hypothesis [59, 63, 138] ISF is produced by secretion across the blood–brain barrier and flows out of the parenchyma along “preferred routes” including periarterial spaces, white matter tracts, subependymal spaces and possibly perivenous spaces. Solute movement within interstitial spaces is by diffusion. The velocity of the flow within the interstitium is too small to produce observable movements of solutes. **b** In the glymphatic hypothesis [11, 36] CSF enters the parenchyma via periarterial routes, flows into the interstitial spaces where it mixes with ISF and sweeps solutes to perivenous spaces. ISF flows out of the parenchyma along perivenous spaces. In simple extensions of the hypothesis, outflow may also occur via white matter tracts and subependymal spaces. **c** Scheme based on current evidence of possible processes involved in supply and removal of solutes in the brain parenchyma. Solutes may move in both directions via periarterial spaces and possibly also via perivenous spaces. There may or may not be net inflow along periarterial spaces and outflow along perivenous spaces. There are also other routes for outflow of fluid and efflux of solutes including white matter tracts and subependymal spaces (compare [34]). Fluid flow may be important in efflux of solutes via extravascular pathways but the flow velocity in interstitial spaces is too small to produce observable movements of solutes. Contrary to what is inferred in many figures portraying the glymphatic circulation, e.g. Fig. 1, there is no sweeping of solutes towards perivenous spaces. In all three schemes solutes emerging from the parenchyma by extravascular routes may be delivered directly to lymph or to CSF. From CSF they can leave the brain via lymph or possibly blood flow

Comparisons of the original classical hypothesis for extravascular efflux of solutes, the glymphatic hypothesis as proposed in 2012, and the processes currently under consideration to explain extravascular transport are indicated in Fig. 9.

In obtaining and interpreting the evidence leading to these hypotheses a number of issues need to be considered.

- Data on entry of solutes added to the cisterna magna, ventricles, or intrathecal spaces have repeatedly been used to assess the rate of glymphatic circulation without determining the concentrations in CSF near the sites of entry into the parenchyma. There are three reasons why this practice may lead to unreliable interpretations. Firstly changes in solute influx may reflect primarily changes in solute delivery to the sites for entry into parenchymal periarterial spaces rather than changes in solute penetration into the parenchyma via the periarterial spaces. Secondly the net flux of solute within the periarterial spaces may occur by mixing or dispersion rather than via net flow of fluid. Finally there is no evidence that the inflow via periarterial routes and the outflow via perivenous pathways and other “preferred pathways” are in fact equal as they would be in a simple circulation.
- In most studies, AQP4 appears to be important in facilitating extravascular transport. However, the explanation of its effects is almost certainly not that it facilitates flow by providing a conduit for the flow of water between perivascular spaces and interstitial spaces of the parenchyma. The importance of AQP4 for entry and exit of solutes must lie in somehow facilitating movement of *solutes* including NaCl (see Sect. 5.3). If this occurs at the level of the endfeet, it might be either by its interactions with other proteins or perhaps by a change in the nature of the endfoot barrier, e.g. changes in endfoot volume and the size of the gaps between the endfeet.
- Some investigators propose that some of the solutes cleared from the parenchyma by extravascular transport are delivered to meningeal lymphatics without first being added to CSF in the subarachnoid space. The mechanism is still unknown including the route by which the solutes destined for lymphatics leave the parenchyma. Both perivenous and periarterial routes are possible, as may be other alternatives, though the available evidence is weighted against a primarily perivenous pathway. Solutes can enter meningeal lymphatics from CSF, but these lymphatics may or may not be important for removal of a significant volume of CSF (see Sect. 5.6). It is difficult to reconcile the proposal that most of the extravascular efflux from the parenchyma occurs by directing most of a glymphatic circulation to meningeal lymphatics [34] with the observation in Sect. 5.5.2 that the flow required appears to be similar to total CSF production by the choroid plexuses. One possible resolution is that there is substantial secretion of ISF across the blood–brain barrier. Further investigation is required.

## Conclusions

To explain how hydrophilic solutes that are unable to cross the blood–brain barrier could be cleared from the brain parenchyma, a description of extravascular transport was put forward in the 1970s [59, 63, 138] (see Fig. 9a). This classical hypothesis proposed a combination of fluid secretion across the blood–brain barrier, diffusion in interstitial spaces and extravascular flow outwards along “preferred routes” including periarterial and perivenous spaces, white matter tracts and subependymal spaces. Considered alone each part of the classical hypothesis is still consistent with current evidence, but as a whole it is clearly not a complete description. For example it does not explain the observed inward movements of solutes from CSF. In 2012, partly to remedy this defect, the glymphatic hypothesis [11, 36] (see Fig. 9b) was introduced to explain how entry and exit of solutes and their movement through the parenchyma could occur. In this alternative scheme instead of there being secretion of fluid across the blood–brain barrier there was fluid flow into the parenchyma along periarterial routes; instead of diffusion in the parenchyma, solutes were swept through the parenchyma by the flow; and, instead of flow outwards via a variety of “preferred routes”, flow outwards was along perivenous spaces. There is a beautiful simplicity to this idea of transport of hydrophilic solutes by a circulation of CSF into, through and out of the parenchyma. Unfortunately current evidence concerning certain aspects of extravascular transport of solutes is not consistent with this simple glymphatic hypothesis.

The glymphatic hypothesis provides no explanation for the observed periarterial efflux of solutes from the parenchyma and, in its original version, invokes perivenous efflux, a process for which there is at present little if any evidence. Furthermore, the outward flow required to account for measured solute clearances of extracellular fluid markers is as large as the total rate of production of CSF by the choroid plexuses whereas the rate of fluid circulation calculated from measured solute influx from CSF into the parenchyma is much less. Finally, the flow through the interstitial spaces of the parenchyma that would be required to sweep solutes more rapidly than would occur by diffusion is more than an order of magnitude larger than the rate of CSF production. All evidence available either in the 1970 s or later supports or is at least consistent with the idea that solute movements through the interstitium within grey matter are governed by diffusion, a process that takes solutes down their concentration gradients be they towards venules, arterioles, white matter or subependymal spaces.

However, in both the classical hypothesis and the glymphatic hypothesis, some form of sweeping of solutes by flow or mixing in addition to diffusion is needed within “preferred routes”, but it is not clear that solutes in these spaces are being carried by a net flow. Thus, it has not been established that under normal conditions there is a connection between solute efflux and either blood–brain barrier secretion or periarterial fluid inflow. There is still an urgent need for more research into the underlying transport mechanisms.

On present evidence, the most likely scenario (see Fig. 9c) is that there is net CSF flow inwards along extramural arterial perivascular spaces as proposed in the glymphatic hypothesis by Iliff et al. [11] but this is considerably smaller than they envisaged. Furthermore, either because the inward periarterial flow is too small or because there is a separate path for solute efflux, the inward flow does not prevent periarterial solute efflux. There is net ISF outflow, some along white matter tracts and subependymal spaces and some via perivascular spaces. Some of the outflow is directed to the ventricles, some to subarachnoid spaces and some to lymphatics in the meninges running alongside the dural sinuses or at the base of the brain. This outward flow balances the combination of any net periarterial inflow, possible net inflow across the blood–brain barrier reflecting secretion of ISF, and production of water from metabolism (compare [69]). The relative importance of perivenous spaces, intra- or extramural periarterial spaces, white matter tracts and subependymal spaces as routes for solute efflux and fluid outflow is still under consideration. Any net flows of fluid in parenchymal perivascular spaces are superimposed on other forms of convection. In the same way that large back and forth movements of CSF with much smaller net CSF flows have been seen in the cerebral aqueduct and the foramen magnum, there may be similar fluid movements but on a smaller scale in the parenchyma along the perivascular routes. These may augment movements of solutes down their concentration gradients along these routes. (Even the extreme proposal of Bradbury et al. [138] that the spaces are occasionally emptied and refilled should still be considered.) It is the combination of diffusion (dominant near brain surfaces), the net flows, other forms of convection and possibly active vasomotion that accounts for both the extravascular delivery of solutes to and the extravascular removal of solutes from the parenchyma. It is possible that both influx and efflux occur along both arterioles and venules (compare with Fig. 8 in [3]). Efficient efflux of wastes along the various routes will maintain the gradients for diffusion out of the parenchyma much as envisaged many years ago by Bradbury and Cserr [59, 63] (compare [109]).

Both the classical hypothesis and the glymphatic hypothesis in its original form fall short of explaining adequately how solutes and fluid pass through the brain parenchyma. Neither can account fully for the processes involved and each requires modifications and changes. The classical hypothesis does not mention periarterial fluid inflow and solute influx. The glymphatic hypothesis in its original form talks of flow rather than diffusion as the dominant process for transport through the interstitial spaces of the parenchyma and does not acknowledge a role for ISF secretion across the blood–brain barrier. Furthermore it does not include a role for the “preferred routes” for solute efflux other than perivenous routes. Both hypotheses need modification to consider forms of convection other than net flow. Despite their shortcomings, both hypotheses have provoked useful argument. Indeed much of the increased attention paid in the past decade to extravascular elimination of toxic wastes and to the mechanisms of extravascular solute transport can be traced to the stimulus provided by the glymphatic hypothesis.

## Data Availability

No new data are reported in this review. There is no data to share.

## Abbreviations

AQP4: Aquaporin 4
CSF: Cerebrospinal fluid
FITC-dextran: Fluorescein isothiocyanate dextran
gadospin: Polymeric gadolinium chelate contrast agent used in MRI
gadobutrol: Gadolinium based contrast agent used in MRI
Gd-DOTA: Low molecular weight gadolinium based contrast agent used in MRI
Gd-DTPA: Gadolinium-diethylenetriamine pentaacetic acid contrast agent used in MRI
iNPH: Idiopathic normal pressure hydrocephalus
ISF: Interstitial fluid
MRI: Magnetic resonance imaging

## Footnotes

1 The term “extravascular” is not meant to exclude intramural perivascular routes; “extra” implies, only that the routes are separate from the intravascular pathway for blood flow. Perivascular routes are extravascular routes that follow the blood vessels.

2 Iliff et al. have provided a movie, available at https://stm.sciencemag.org/highwire/filestream/194273/field_highwire_adjunct_files/1/Movie_1.avi, of these events for a 40 kDa FITC-dextran. This contains images of an approximately 700 μm x 700 μm field at 0 60 and 120 μm depths repeated at 1 min intervals for 34 min. The artery shown traversing the field appears to fill with the dextran along its length in a single time interval. It then spreads continuously outwards over the rest of the 34 min period. Passage along several penetrating arteries is apparent within 15 min and increases for the rest of period.

3 The extensive experiments on time course of efflux of albumin, dextrans and polyethylene glycols conducted by Cserr and associates (see e.g. [59, 62, 63]) were all performed under barbiturate anaesthesia. Groothuis et al. [214] first drew attention to the fact that with different anaesthetics, in particular ketamine/xylazine, efflux was much faster. The results obtained by Iliff et al. [11] and Xie et al. [37] are consistent with those of Groothuis et al.

4 If flow from periarterial space into parenchyma is partially blocked, volumes and pressures throughout the pathway will change until the flow into the parenchyma is increased and the flow through and out of it decreased until the flows are again in balance. An initial reduction in flow across the glial endfoot layer will lead after adjustments to a smaller reduction because the pressure difference across the rate limiting step will be larger with net flow again the same at all stages of the pathway [3].

5 The observation in one study [157] of a net counterflow of nanoparticles still requires explanation. van Veluw et al. added nanoparticles locally to the CSF by laser induced extravasation while all other groups injected their markers into the cisterna magna. van Veluw et al. suggested that the forward movement seen in many studies was a result of raised pressure in the cisterna magna caused by the infusion. However subsequently Raghunandan et al. [102] used simultaneous infusion and withdrawal of fluid from the cisterna magna to inject the microspheres and avoid the increase in intracerebral pressure and obtained the same results as Mestre et al. [45, 89].

6 This time sequence of concentrations on the ventral and dorsal surfaces of the brain in both experimental animals and humans is evidence that arachnoid villi leading to the saggital sinus are not the main efflux pathway for CSF. Elimination of markers will produce lower concentrations near the sites of elimination, i.e. these are not at the dorsal surface. This point will be considered in greater detail elsewhere.

7 Eide et al. [215] have reported on concentrations of gadobutrol in brain and blood after injection into lumbar CSF. “The peak concentration in blood (at about 10 hrs) preceded by far peak tracer enhancement at MRI in extra-cranial lymphatic structures (at about 24 hrs) as shown in previous studies, indicating a major role of the spinal canal in CSF clearance capacity.“ This emphasizes yet again that distribution of markers for extracellular fluids by CSF is an important factor in their delivery to the brain parenchyma.

8 Mestre et al. ([122], see also [186]) have asserted that any acute procedure involving injection of a solute into the parenchyma would lead to a global suppression of glymphatic flow. This would affect periarterial solute fluxes. However, while such an effect may explain why early workers failed to note periarterial influx, it does not alter that they did observe periarterial efflux at a rate faster than could be explained by diffusion alone (see Sect. 5.2.2).

9 Ma et al. [53] quite rightly point out that within an hour entry of markers into the first millimeter or so of parenchyma can occur to a significant extent by diffusion across the glia limitans and through the interstitium. In the human studies entry into the parenchyma appears to take hours so diffusion would suffice for superficial regions. However, the evidence for penetration via perivascular spaces is not the time taken in superfical cortex but rather that the markers reach deep regions of the cortex for which diffusion would be too slow.

10 Bakker et al. [44] suggest that whether fluxes and flows in the periarterial spaces are inwards or outwards could be determined by pressures produced by the infusion of markers, with this being regarded as more likely with infusions into the interstitium than into the cisterna magna. It is undoubtedly true that flows during infusions may be dictated by the infusions for the simple reason that the smallest volumes that can be infused are larger than the dimensions of the intended sites of infusion. (1 µL is 1 mm^3^ which is a large volume in the interstitium of a mouse). However, as Cserr et al. and many others since have been aware this effect is likely to be transient provided the total volume infused is kept below 1 µL. Infusions of total volumes up to even 10 µL (see e.g. [11]) into the cisterna magna of a mouse may be acceptable *after the infusion is stopped* for the simple reason that there are low resistance pathways for the infused fluid to be distributed over a much larger volume than that of the cisterna magna.

11 Ghersi-Egea et al. [216] found that when I^125^-amyloid-β was injected into a lateral ventricle of a rat it was rapidly cleared into blood. with ~ 70% eliminated from the brain within 30 min. Penetration into the parenchyma was limited to a thin layer adjacent to the ventricles, as expected if the amyloid that penetrated was rapidly cleared into blood capillaries. (Entry into the choroid plexuses was also seen.) After ~30 min the amyloid-β remaining in the brain was primarily bound to pial arteries and arterioles. These results demonstrate clearly that there is transport of amyloid-β across the blood–brain barrier and that the peptide binds to arterial walls. Ghersi-Egea et al. suggest that amyloid-β reaches arterial walls within the parenchyma by penetration along peri-arterial spaces from the subarachnoid spaces. Albargothy et al. [129] have observed that amyloid-β added to CSF does indeed reach arterial walls within the parenchyma, but note that after addition it is first seen on the outer surface of the walls and only subsequently is seen at locations where endogenous amyloid-β is deposited. Ghersi-Egea’s results therefore reinforce the importance of the intravascular route for elimination of amyloid-β but do not address the question of the location of the extravascular route of elimination of amyloid-β from the parenchyma which all are agreed does exist.

12 Infusions of 0.5 µL as used by Szentistvanyi et al. [62] are more than 0.1 % of the total extracellular fluid volume of the rat brain but are added in a small local region. Furthermore the infusion rate, 63 nL min^-1^, is about 5 % of the CSF production rate but the induced flow is all in a local region. Infusions inevitably force flows. However, the transients in pressure and flow at the onset of infusion and the persistence of raised pressure and flow after the end appear not to have not been modelled explicitly. The analysis that has been used in the studies on convection-enhanced delivery [144] assumes that the excess pressure distribution is created rapidly (i.e. in seconds or at most a few minutes) at the onset of an infusion and decays similarly rapidly at its end. The decay would be “instantaneous” in the case for infusion into a region that could be described as an incompressible fluid. An experimental suggestion that pressures resulting from injection or infusion decay quickly is that reflux occurs along the low-resistance track left behind when an injection needle or pipette is withdrawn immediately at the end of an infusion but not if it is left in place for two minutes before withdrawal (see e.g. [8]).

13 This is the total systolic expansion which is made up of a larger expansion of arteries and some contraction of veins, mainly venous sinuses [217].

14 A 50 % change in periarterial space width along penetrating arteries isn’t as improbable as it may first sound because it may correspond to a much smaller change in the dimension of the artery. For instance, if the artery is cylindrical with radius r and the periarterial space is the gap between that cylinder and a rigid cylinder with radius 1.1r, i.e. with width 0.1r, then a 50 % reduction in width would require only a 5 % increase in the radius of the artery from r to 1.05r. However even this is still much greater than the changes thought to occur.

15 When interpreting Fig. 6 it is necessary to recall that in the parenchyma the extracellular space is only about 20 % of the total volume. Thus if a marker for extracellular fluid had the same concentration in ISF and CSF, the concentration in the tissue (a weighted average of extracellular and intracellular concentrations) and hence the brightness in the image would be only 20% as high as in CSF. It is the data towards the end of the sampling period that indicates persistence of gadobutrol in the tissue and hence that efflux is slow.

16 Conversely the flows could in principle be greater than indicated by the entry of markers if the markers were subject to sieving (as if being held back by a filter) compared to the most abundant solute, NaCl. However, dextrans of a range of sizes enter at the same rate which is thought to exclude this possibility [11].

17 The water permeability of lipid bilayers can be substantial depending upon their composition and could account, for instance, for the water permeability of the endothelial cells of the blood–brain barrier (see Sect. 4.3.6 in [1] and Sect. 5.1 in [2]). Indeed specialized membranes are needed where it is necessary to *prevent* water movements, e.g. in the luminal membranes of epithelial cells in the thick ascending loop of Henle in the kidney. Aquaporins or some other means for increasing water permeability of membranes are required to allow: regulated, large net fluxes as in renal cortical collecting tubules; modest fluxes but with small osmotic gradients as in renal collecting ducts; or transfer of small amounts of water but in a very short time. None of these conditions are obviously applicable to water movements between perivascular spaces and the parenchyma under physiological conditions. For early surveys of locations where aquaporins are important see [218, 219].

18 Even a very small osmotic gradient produced by flow-induced concentration changes in the solutes, so-called concentration polarization, could overshadow any conceivable hydrostatic pressure difference between the perivascular spaces and the interstitium, e.g. a 1 mOsM difference can nullify the effects of a hydrostatic pressure difference of 19 mmHg (see [3] for discussion). This does not contradict the importance of AQP4 in cytotoxic oedema where the osmotic gradient drives the water flow and the flow-induced concentration changes would have to offset a large fraction of the initial gradient [167].

19 AQP4 knockout in mice does reduce unidirectional fluxes of water from blood to parenchyma [220, 221], the AQP4 inhibitor TGN-020 decreases the rate of entry of H^17^_2_O into the parenchyma [222], and the AQP4 facilitator TGN-073 increases the rate of exchange of water molecules in several regions of the brain [223]. However, in these MRI studies just as with radiotracer studies, it is not possible to infer effects on net fluxes of water from observed effects on the very much larger unidirectional or exchange fluxes (see [3] for discussion in relation to blood–brain barrier transfers of water). As noted in the preceding footnote any net flow of water across the endfoot layer in physiological conditions will be determined in large measure by the movement of the osmotically active solutes, primarily NaCl.

20 The development of cytotoxic oedema in the first few hours following middle cerebral artery occlusion depends on production of new osmoles by catabolism within the parenchyma and on transfer of NaCl from CSF and blood. Much of the NaCl must cross the endfoot layer. This will be considered in a subsequent review on oedema.

21 The observation by Bedussi et al. [126] of clearance by fluid flow from the striatum towards the ventricles was based on measurements made during the infusion of fluid. Infusion directly into the parenchyma can produce flow through the tissue. This strategy is the basis of convection enhanced delivery of drugs, see e.g. [224–227].

22 Based on the pattern and speed of spread of gadoteric acid (Gd-DOTA), Koundal et al. [103] asserted that “advective [meaning associated with flow] transport dominates in CSF while diffusion and advection both contribute to GS [glymphatic system] transport in parenchyma.” However, in their analysis they combined the fluid in perivascular spaces of vessels running through the subarachnoid spaces together with CSF and similarly combined the fluid within perivascular spaces of parenchymal vessels together with the rest of the ISF. Thus in their analysis the flow within the “parenchyma” could have been restricted to perivascular spaces with diffusion dominant within the interstitial spaces (compare [45, 109]). Similarly, Valnes et al. [124] concluded based on their observations of long-term movements of gadobutrol that “diffusion may not be the only mechanism governing transport [in the parenchyma]”. This again is consistent with the idea that there is diffusion in the interstitial spaces and some form of convection in perivascular spaces. Croci et al. [123] reanalyzed the gadobutrol MRI results of Ringstad et al. [116] and again showed that diffusion alone cannot explain the results. However, given that they “emphasize that each model represents a homogenized velocity field averaged over physiological structures” it remains possible, even probable, that the assumption of flow or mixing being restricted to perivascular spaces would allow the data to be fitted. It should be remembered that in all these studies the spatial resolution of the experimental technique that provided the data being analysed was not adequate to separate movements in perivascular spaces within the parenchyma from those in the interstitial spaces. There is some hope that very high field measurements with MRI instruments for use with small animals may make such resolution possible in the future [128].

23 From the effects seen by Iliff et al. [15] and that reported in Fig. 7 of [122] suppression might be better called reduction as it is not complete. Iliff et al. observed an ~25 % reduction in efflux of markers following traumatic brain injury. The effect on influx of a cisternally injected marker was markedly reduced ipsilateral to the injury, but this may reflect changes in the distribution of CSF as well as changes in penetration into the parenchyma. In their Fig. 7 Mestre et al. report a 40 % to 50 % reduction in global influx of a cisternally injected marker, but again this may reflect effects on the distribution of CSF flow in the subarachnoid spaces. The proposal that a local disturbance produces a suppression of glymphatic transport over a large portion of the brain may be closely related to the issue of whether the variations of periarterial flows and fluxes tied to the cardiac cycle are related to local changes in arterial diameter or to the more general changes occurring throughout the brain that are thought to drive the cyclic flows of CSF through the aqueduct and foramen magnum.

24 The computational descriptions of flow in the interstitium have assumed that a fluid with the viscosity of a simple electrolyte solution is constrained to move through the spaces between the cells. This may substantially overestimate the flows. The interstitial fluid contains extended macromolecules (see e.g. [228]) and is perhaps better thought of as being a gel, i.e. a matrix containing a free fluid. One common example of such a gel is agar, another is Matrigel [195]. The matrix of a gel may have only a small effect on diffusion while still greatly reducing flow (see [229, 230], Sect. 2.3 in [3], and [54]). The nature of the extracellular fluid and matrix is considered in [34, 127, 191, 231]. However, when reading those accounts, it should be remembered that while the presence of macromolecules in brain interstitial spaces is important in maintaining their patency those macromolecules also impede flow (i.e. for the same dimensions of the interstitial spaces the resistance is higher if there are macromolecules than if there were just water and low molecular weight solutes like NaCl). This makes even more secure the conclusion [171, 192] that flow at a significant rate in the interstitial spaces of the non-oedematous brain requires pressure gradients that are impossible to achieve (see footnote 8 in [2]).

25 This velocity is a “superficial velocity” defined as the flow per unit area of tissue without regard to whether on a microscopic scale all of the area is actually accessible for the flow. In the brain about 4/5ths of the area is occupied by cells and hence inaccessible. The actual velocity of the flow within the interstitial spaces would therefore be about 5 times higher. For further discussion see [193, 199] and for discussion of the same and related issues for the diffusion constant see [185, 232]).

26 In general the flow between sources and sinks of flow in a region is the average flow velocity perpendicular to a surface that separates the sources from the sinks times the area of that surface. In Ray et al’s model (see Fig. 7) a convenient surface is the midplane between planes containing the arterioles and venules. Because the area of the midplanes in a gram of the model tissue is the volume of tissue, ~ 1 cm^3^ g^-1^, divided by the unit cell spacing, 250 μm, this area equals 40 cm^2^ g^-1^ and the flow through the tissue would need to be at least the minimum average flow velocity, 10 μm min^1^, times the area, 40 cm^2^ g^-1^ which is 40 µL min^-1^ g^-1^ [199]. In a rat with a 2 g brain this would be a total flow of more than 80 µL min^-1^. For comparison, CSF production in a rat is about 1.2-1.8 µL min^-1^ [233–235] and thus the required flow is more than 80/1.8 = 44 times larger than the CSF production rate. The comparable hypothetical glymphatic flow scaled for a human with a 1400 g brain would be 56 mL min^-1^ to be compared with a CSF production of 0.35 mL min^-1^, i.e. 160 times greater. The simple calculation in the preceding paragraph assumes that CSF flow is uniformly directed to all portions of the brain which is of course a gross simplification. However, the errors introduced are such that the conclusion is even more secure. Thus grey matter will receive less per gram of tissue than the average for the brain as a whole (CSF production divided by brain mass) even if only because most CSF never approaches grey matter (see Sect. 5.2.5).

27 Ray & Heys [194] have presented an extensive, thoughtful review on fluid flow in the tissues of the brain. They come to the conclusion that flow in the interstitial spaces is sufficient to affect transport of macromolecules but not small solutes. Because the discussion here makes use of their model for the vasculature, the difference in conclusions between the account here and theirs requires some comment. The partial disagreement arises from a difference in opinion as to the likely flow velocity in the parenchyma. They consider a range from 10 to 100 μm min^-1^. However, this range corresponds to impossibly large flows. Using the entire CSF production of ~1.5 µL min^-1^ distributed uniformly over the 2 g brain of a rat, the velocity would be only 0.19 μm min^-1^ (see next section). This much lower value is consistent with the expectations of Jin et al. [171] and Holter et al. [192]. It should be noted that even this value for the velocity may be an overestimate as the net flow may be smaller than assumed. In terms of Fig. 3 in [194], based on a characteristic length of 240 μm for the distance to a perivenous space, 0.19 μm min^-1^ would yield a much larger characteristic time for interstitial advection of ~1250 min, implying that for all molecular sizes considered by Ray & Heys interstitial transport would be dominated by diffusion. Ray et al. [193] cited the figure of 10.5 μm min^-1^ reported by Rosenberg et al. [60] for the velocity of flow towards the ventricles in white matter as indicating that 10 μm min^1^ is reasonable for the velocity of flow in grey matter. For two reasons this comparison is not very helpful. Firstly the matter of geometry: 10 μm min^-1^ across the area of white matter leading to the ventricles corresponds to a much smaller flow than 10 μm min^-1^ across the area available for flow between arterioles and venules within grey matter. Secondly flow aligned with the direction of fiber tracts in white matter is expected to encounter much less resistance than flow through the confined and tortuous spaces of grey matter.

28 If a hydrophilic extracellular fluid marker is at the same concentration throughout the extracellular fluid in a tissue with perivascular spaces (possibly including basement membranes), the amount of marker per unit volume of tissue will be higher in the perivascular spaces simply because the volume fraction of extracellular space is ~5x higher. It is not clear whether or not this effect has been taken into account in the interpretation of images. This may be important for fluorescence images (see e.g. Figure 3G in [11], Fig. 2f right hand frame in [196]) that appear to show higher concentrations of makers adjacent to venules.

29 If the mechanism for efflux of an extracellular fluid marker is outflow of fluid containing the marker at the same concentration as in the interstitial fluid, then the clearance for that marker will be equal to the flow (see Sect. 3.2 in [2]). If the concentration of the marker in the outflowing fluid is less, then the flow must be greater than the clearance. Thus the clearance is likely to underestimate the flow required. (Higher concentration would require active transport—i.e. a more complicated theory.) In mice and rats for markers like sucrose, inulin and albumin the clearances are ~ 1 µL min^−1^ g^−1^ (see Table 1 in [2]) and thus in a rat with a 2 g brain the total required outflow is 2 µL min^−1^ which is larger than estimates for the total CSF production by the choroid plexuses (see footnote 26). Half-lives for elimination are longer in humans than in rats and mice (10 or more hours in humans asleep for part of the observation period [117, 166, 236] compared with ~ 2 h in rats that were anaesthetized [214]), and thus the clearances in humans for elimination by extravascular pathways may be about 5 fold smaller, ~ 0.2 µL g^−1^ min^−1^. For a 1400 g human brain this would be 0.28 mL min^−1^ which is somewhat smaller than CSF production rate, ~ 0.35 mL min^−1^.

30 While direct outflow of ISF to lymph is an attractive proposal (see discussion in this section and in Sect. 5.2.2), neither the citation [237] given by Rasmussen et al. as the source of the 80 % figure nor subsequent publications from Cserr’s group (see e.g. [62, 96, 138] in fact support this proposal. All of their data can also be explained by ISF outflow from the parenchyma into CSF in subarachnoid spaces much of which is then directed to lymph without returning to the cisterna magna, e.g. via the cribriform plate [96]. The proportions of ISF outflow that mixed with CSF in their experiments cannot be determined from their data though they must be at least as large as the proportions they found in their CSF samples.

31 There is, of course, ample evidence that solutes injected into the parenchyma are delivered to lymph nodes. However, the route for some of this delivery, at least in rats, is via efflux to CSF and then delivery of CSF to lymph via the cribriform plate [62, 209] (see Fig. 8).
